# No Control Genes Required: Bayesian Analysis of qRT-PCR Data

**DOI:** 10.1371/journal.pone.0071448

**Published:** 2013-08-19

**Authors:** Mikhail V. Matz, Rachel M. Wright, James G. Scott

**Affiliations:** 1 Department of Integrative Biology, University of Texas at Austin, Austin, Texas, United States of America; 2 Institute for Cell and Molecular Biology, University of Texas at Austin, Austin, Texas, United States of America; 3 McCombs School of Business and Division of Statistics and Scientific Computing, University of Texas at Austin, Austin, Texas, United States of America; University College London, United Kingdom

## Abstract

**Background:**

Model-based analysis of data from quantitative reverse-transcription PCR (qRT-PCR) is potentially more powerful and versatile than traditional methods. Yet existing model-based approaches cannot properly deal with the higher sampling variances associated with low-abundant targets, nor do they provide a natural way to incorporate assumptions about the stability of control genes directly into the model-fitting process.

**Results:**

In our method, raw qPCR data are represented as molecule counts, and described using generalized linear mixed models under Poisson-lognormal error. A Markov Chain Monte Carlo (MCMC) algorithm is used to sample from the joint posterior distribution over all model parameters, thereby estimating the effects of all experimental factors on the expression of every gene. The Poisson-based model allows for the correct specification of the mean-variance relationship of the PCR amplification process, and can also glean information from instances of no amplification (zero counts). Our method is very flexible with respect to control genes: any prior knowledge about the expected degree of their stability can be directly incorporated into the model. Yet the method provides sensible answers without such assumptions, or even in the complete absence of control genes. We also present a natural Bayesian analogue of the “classic” analysis, which uses standard data pre-processing steps (logarithmic transformation and multi-gene normalization) but estimates all gene expression changes jointly within a single model. The new methods are considerably more flexible and powerful than the standard delta-delta Ct analysis based on pairwise t-tests.

**Conclusions:**

Our methodology expands the applicability of the relative-quantification analysis protocol all the way to the lowest-abundance targets, and provides a novel opportunity to analyze qRT-PCR data without making any assumptions concerning target stability. These procedures have been implemented as the MCMC.qpcr package in R.

## Introduction

Real-time quantitative PCR [1] is a gold standard for quantifying the abundances of nucleic acid targets (DNA or RNA molecules of a particular sequence). One of its most common implementations, quantitative reverse transcription-PCR (qRT-PCR), is a gene expression quantification method extensively applied to test specific hypotheses suggested by genome-scale approaches (microarrays or RNA-seq), as well as for analyzing diagnostic gene expression signatures in studies ranging from medicine to ecology. Despite the widespread use of qRT-PCR, the data processing and statistical analysis procedures are still in flux, with many alternative approaches coexisting in the literature and new methodologies continuously developed. One of the earliest qRT-PCR analysis methods still very commonly used is relative quantification using the 2^−ΔΔ*C*T^ (“delta-delta Ct”) method [2]. It compares pairs of samples to see whether a target gene became more or less abundant relative to the control gene, the expression of which is assumed to be constant. This approach is attractive because of its mathematical elegance and the ability to correct for unequal amounts of biological material (“template loading”) between samples by using the control gene as an internal standard. As a disadvantage, however, it relies on pairwise comparisons of samples and therefore makes it difficult to handle more elaborate experimental designs, particularly involving interactions between factors [3]. Furthermore, Pfaffl et al [4] pointed out an additional complication: the need to account for the difference in efficiency of amplification of the control and target gene. This problem was addressed by replacing the original 2^−ΔΔ*C*T^ equation with a four-story formula incorporating the efficiencies of PCR for control and target gene [4]. These efficiencies (amplification factors per single thermal cycle) are typically determined by qPCR analysis of dilution series [4]–[6], although other methods, based on the analysis of individual product accumulation curves, have been suggested [7]–[10].

The need to base qPCR analysis on dilution series data made relative quantification practically equivalent to the other flavor of qRT-PCR analysis, absolute quantification [11]. Under this approach, the raw Cq (“cycle of quantification”) values are transformed into concentrations of the target per qRT-PCR reaction, based on the calibration curve constructed across a series of known target concentrations. The efficiency of amplification is implicitly taken into account during this conversion. Since in qRT-PCR the knowledge of the absolute target amount is usually not as important as the knowledge of its variation across samples, so-called relative calibration curves, created by diluting an arbitrary amount of target, are often used [12], [13]. These relative calibration curves provide essentially the same information as the calibration curves for determining PCR efficiency [4]. To account for variation in template loading across samples, the procedure called normalization is performed where the inferred target amounts are divided by the abundance of a control gene. In this way, all the target abundances become expressed as fold differences relative to the abundance of the control gene [14]. However, hardly any one gene remains perfectly stable [15]. Vandesompele et al [16] proposed that more accurate normalization could be achieved by using multiple nearly-stable control genes: in this case, the target abundances are divided by the geometric average of the control gene abundances. The same paper also introduced a non-parametric method, geNorm, for identification of the most stable control genes based on covariance across samples, which quickly became a standard in the qRT-PCR field [17]. Another commonly used method for identifying stable genes is the parametric NormFinder algorithm [18], which makes use of the fact that the log-transformed qRT-PCR data satisfy the normality criterion [19] and uses moments equations to calculate the stability of each gene independently of other genes. Following normalization, several authors used further parametric approaches such as ANOVA [6],[19] and linear mixed models [20]–[23], applied on a gene-by-gene basis, to achieve maximum versatility in analysis of complicated designs. The workflow involving correction for amplification efficiency followed by multi-gene normalization and gene-by-gene analysis with t-tests, ANOVA, or linear modeling represents the current consensus of qRT-PCR data processing [24].

We would draw an analogy with the literature on data analysis for DNA microarrays. Here, it has been repeatedly argued that the joint analysis of the whole dataset is more appropriate. Such an approach can borrow information across multiple genes, improve the precision of gene-specific estimates, and properly account for complex experiment designs, as well as both biological and technical replication [25]. In the qRT-PCR field, this approach has been explored by Steibel et al [3], who developed a parameter-rich linear mixed model to jointly estimate all the gene-specific effects from non-normalized qRT-PCR data. The most notable feature of this model is its inclusion of unobserved random effects, common to all genes in a sample. These random effects account for unequal template loading between samples, thereby achieving a functional equivalent of normalization.

Despite the attractiveness of this approach, three major issues remained unresolved. First, the approach of Steibel et al. disregards heteroscedasticity, or the increase in sampling variance at the lower end of target abundances. In this respect it is similar to essentially all existing qPCR analysis pipelines: the statistical model ignores the discrete nature of the amplification process. This heteroscedasticity arises because qRT-PCR is fully capable of amplifying just a few target molecules within each trial [26], [27]. It thus becomes prone to Poisson-like “shot noise” [28]. Second, the method cannot easily derive information from PCR trials in which the sample failed to amplify simply because it contained zero target molecules. Finally, no solution was provided to directly include information about control genes into the model-fitting process.

Here, the mixed-modeling approach of Steibel et al is extended to account for all these issues. First, a generalized linear mixed model based on the Poisson-lognormal distribution replaces the original Gaussian model. This properly handles zero counts, as well as the shot-noise variance associated with low-abundant targets. Second, the model fitting process involves a Bayesian MCMC sampling scheme, and can directly incorporate information about control genes in the form of priors. Our implementation of the method leverages the MCMCglmm package in R [29] and is presented in the form of a specialized R package, MCMC.qpcr.

## Results

### Motivating example

The dataset that was chosen for re-analysis addressed the effects of heat-light stress and recovery in a reef-building coral *Porites astreoides* [23]. Briefly, eight individual colonies of the coral were fragmented into 4 pieces each and allowed to acclimate in common benign conditions for four days. On the fifth morning, two fragments of each colony were placed into a stressful environment (elevated heat and light). At midday, when the stress intensity was the highest, one stressed fragment and one control fragment (remaining in the benign conditions) were sampled, representing the first sampling timepoint. In the end of the same day, the second stressed fragment was put back into the benign environment for recovery. This fragment, along with the remaining control fragment, was sampled at midday on the following day (the second timepoint). Expression of 15 genes, 5 of which were putative control genes, was assayed by qRT-PCR on LightCycler 480 (Roche) with SYBR-based detection, corrected for amplification efficiencies, normalized by the 3 genes that proved to be most stable according to geNorm test [16], and analyzed using linear mixed models applied on a gene-by-gene basis [23].

This dataset is interesting from the analytical standpoint because of three reasons. First, one of the main effects of interest is the interaction term, Condition:Timepoint, describing the gene regulation in coral fragments that were first stressed and then allowed to recover. Evaluation of the interaction term necessitates the use of linear models or ANOVA rather than non-parametric methods or pairwise t-tests [3]. Second, the dataset includes an important random factor: the identity of the coral colony from which the experimental fragments were obtained. This factor accounts for variation in the baseline levels of gene expression between individual corals, and prompts the use of a linear mixed model rather than a simple linear model. Finally, several genes were so low-abundant under some conditions that they became undetectable in a considerable number of trials, precluding the straightforward use of log-transformation typical of qRT-PCR analysis [16], [19].

### Poisson-lognormal mixed models

The standard practice for qRT-PCR analysis is to analyze each gene individually, using control genes to estimate the required normalization factors. In contrast, we build a hierarchical model that can be used to jointly estimate the effects of experimental treatments on the expression of all genes. Under such an approach, control genes can sharpen estimates of model parameters, but are not strictly necessary, as all normalization happens within the model.

In this respect, our approach is similar to that proposed by Steibel et al [3], who model the cycle of quantification (*C_q_*) using linear mixed models (LMMs) with Gaussian errors. Since *C_q_* is proportional to the negative logarithm of a gene's initial transcript copy number, this assumption implies that copy number (an integer quantity) is being modeled with a log-normal distribution.

Our approach differs in that we directly model the initial copy number using generalized linear mixed models (GLMMs) with Poisson-lognormal errors [30]. This is a very natural way to model multiplicative fold-changes. It is also more appropriate for count data than a log-normal model, and can flexibly accommodate a wide range of mean-variance relationships. Moreover, unlike the Gaussian model for *Cq*, it also gleans information from samples that fail to amplify (*Cq* = ∞), as these correspond to counts of zero.

A third advantage of our model is that it naturally accounts for the so-called “shot noise” that arises from the discrete nature of PCR amplification. Shot noise refers to Poisson-like fluctuations that become discernible when the number of target molecules is small enough so that such fluctuations are the dominant source of variability after signal amplification. To see why this occurs for weak signals, observe that if the true number of target molecules in a sample is Poisson distributed, then the absolute magnitude of shot-noise variation grows like the square root of the expected number of molecules. This is much slower than linear growth, meaning that the relative contribution of shot noise decreases and the signal-to-shot-noise ratio increases as the expected number of counts gets larger. This explains why shot noise is more frequently observed when amplifying samples with very few target molecules, as we illustrate experimentally below.

In describing our model, we use the following subscript conventions:

*g*: gene
*i*: level of a treatment condition
*j*: level of a grouping variable, e.g. block, plot, genetic line, etc.
*k*: biological replicate (a single RNA sample)
*r*: technical replicate.

Thus *y_gijkr_* is the count (initial transcript copy number) for gene *g* under treatment *i*, group *j*, sample *k*, and technical replicate *r*. Our model assumes that *y_gijkr_* arises from a Poisson-lognormal distribution:
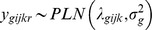
where PLN(m,v) denotes the Poisson-lognormal distribution with rate parameter m and log-variance v. In our model, the log-variance is gene-specific, and the rate terms involve regressions on both fixed and random effects, as detailed in the subsequent section.

There is no closed-form expression for the density of the Poisson-lognormal, but it may be interpreted as a mixture of Poissons. Specifically, suppose that
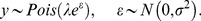
(1)


Then the marginal distribution of *y* is Poisson-lognormal with parameters 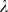 and 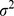. Intuitively, the PLN is similar to the negative-binomial distribution, which can also be expressed as a mixture of Poissons, but which (unlike the PLN) has a closed-form density.

A notable feature of the Poisson-lognormal model is its overdispersion relative to the Poisson: if 
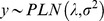
, then
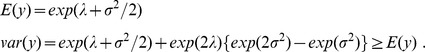



This is important for adequately describing the technical variability of qPCR measurements, which need not match the strict mean-variance relationship implied by the Poisson distribution. Observe that in the limit as the log-variance goes to 0, the model becomes Poisson.

For the purpose of model-fitting, we appeal to (1) and re-write the original model in an equivalent hierarchical form, which facilitates computation via Markov-chain Monte Carlo:
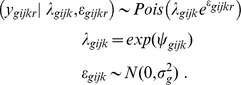



The 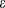 terms, which are mutually independent, appear as random effects in the hierarchical specification. This gives the appearance of a saturated or even non- identified model. But they are best thought of as merely data-augmentation variables that yield a computationally efficient way to recover the original Poisson-lognormal specification (which is neither saturated nor unidentified).

### Structure of the regression model

Our model for log-rate term 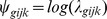 takes the form
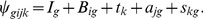
(2)


We describe each component of the model in more detail, along with the priors used for the random effects.

*I_g_* represents a gene-specific intercept. This is always part of the model; it represents the baseline level of each gene's expression under the “reference” or baseline combination of experimental treatments (fixed factors), to which all other treatment effects will be compared. In our coral example, a natural choice for the reference combination is the control condition at the first time point.
*B_ig_* is the fixed effect of treatment *i* on gene *g*. This term derives from the experimental design matrix, and consists of a series of gene-specific fixed effects that we are primarily interested in. We denote this term *B* to signify that it captures the main biological relevance of the study. In our coral example, this corresponds to a series of interaction terms: gene/condition, gene/timepoint, and gene/timepoint/condition.
*t_k_* is a random effect meant to capture unequal template loading (hence *t* ) in biological replicate *k*. Intuitively, this accounts for the reality that, all else being equal, biological replicates may still differ systematically in the transcript copy numbers across all genes due to variation in the gross amount and/or quality of RNA among samples. These effects are modeled with a Gaussian prior where the gene-specific variance is common to all replicates.
*a_jg_* is the gene-specific random effect associated with the *j*th level of some grouping variable, such as block, plot, litter as in Steibel et al [3], or coral colony in our motivating example. Depending on the experimental design, there may be more than one grouping variable, or none at all. These effects are modeled with a Gaussian prior whose variance may, in principle, be gene-specific.
*s_kg_* captures residual variation across different biological samples, assuming that some genes might vary more than others. These effects are modeled with a Gaussian prior whose variance may also be gene-specific.

### Explaining the model using coral example

To make sure we are understood not only by statisticians but also by qRT-PCR practitioners, below we explain in more colloquial terms how the model (2) is constructed for our motivating example.

The model has a single response variable, the transcript count, whose rate is modeled on a log-linear scale. The most basic explanatory variable in the model is ‘gene’, which corresponds to the term *I_g_* in the formula (2) and accounts for different levels of expression between genes. We may express this informally as:
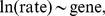
where “rate” refers to the count rate of the Poisson-lognormal model. Intuitively, this is the likely frequency of transcript counts for a particular gene in a particular sample. Such a primitive model, however, would be of little value since in qRT-PCR we are typically not interested in difference in expression between genes, but want to learn how the expression of each gene varies depending on the experimental treatments. To find this out, we augment our model with a series of terms describing gene-specific effects of experimental treatments, corresponding to the term *B_ig_* in the formula (2). In our coral experiment, we have two treatments (or, using linear modeling terminology, factors): Condition with levels “control” and “heat” and Timepoint with levels “one” and “two”, plus their interaction (i.e., we suspect that there might be some Timepoint-specific effects of Condition). This experimental design is incorporated into the model as follows:



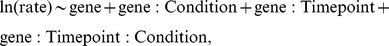
where the colon indicates interaction, essentially standing for “-specific effect of “. The model is fully flexible, not being limited to a particular number of factors, number of levels within each factor, or presence-absence of interactions.

Even though this model specification seems to contain all the terms we want to estimate, we must take care of other important sources of variation that, while being of no real interest to us, must be taken into account to ensure that the model is accurate and powerful. The most important of these is the random effect of the biological replicate (i.e., an individual RNA sample), accounting for the variation in quality and/or quantity of biological material among samples, which corresponds to the term *t_k_* in the formula (2). The designation “random effect” implies that we are not interested in actual estimates of each sample's quality or quantity, but simply want to partition out the corresponding variance. Random effects are imagined as random variables drawn from an underlying distribution the variance of which the model will estimate. In the simplified notation that we use throughout this section, we will list the names of random factors in square brackets, to discriminate them from the factors of primary interest (“fixed factors”) that we discussed before:
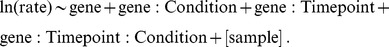



Note that, since the variation in cDNA quality and/or quantity affects all genes in a sample in the same way, this random factor is not gene-specific. The introduction of this random factor into the qRT-PCR model was perhaps the most important innovation in the model of Steibel et al [3].

The experimental design might have involved additional “grouping factors” that are not directly related to the experimental treatments being studied but still might be responsible for a considerable proportion of variation and must be accounted for to achieve more accurate predictions. These factors, if present, would correspond to the term *a_jg_* in the formula (2). For example, the experiment might have involved repeated measurements of participating individuals, partitioning of the experimental subjects between several blocks (plots, tanks) for technical reasons, or measurements of all the effects of interest on different genotypes. The latter is the case in our coral example, where we used 8 coral colonies each split into four clonal fragments that were exposed to our experimental treatments. The grouping factors can be specified in the model as additional random factors; however, in contrast to the *sample* factor, these would be gene-specific since different genes might be affected by the grouping factors differently. In our case, we want to account for possible differences in baseline level of expression of each of our genes between 8 colonies, and we augment our model as follows:
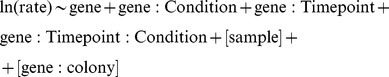



Once again, the model is flexible in the number of grouping factors that could be included.

The two remaining terms that we still need to add are both error terms, accounting for the residual variation that remained unexplained. The first one is specified as a random factor and reflects the unexplained differences between biological replicates (samples), corresponds to the term *s_kg_* in the formula (2). It makes sense to assume that this factor would be gene-specific, i.e., some genes will vary more than others among samples:
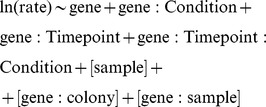



Finally, the remaining unexplained variation would be due to the differences between technical replicates, reflecting the precision of the qPCR instrument used. This term corresponds to the error term *ε* of the general Poisson-lognormal model given by equation (1). We follow Steibel et al [3], who found that the model fit is typically improved when specifying this term as gene-specific:
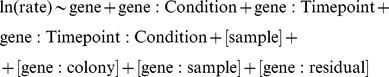



It is important to note that separating variances due to *gene:sample* from *gene:residual* is only possible when the dataset contains technical replicates (which, ideally, it should); otherwise the variances collapse into a single term *gene:residual*.

### Specification of priors

For fixed factors involving genes that are not designated as control genes, a diffuse normal prior is used, with mean  = 0 and very large variance (10^8^) [29]. For control genes, we want to specify that they should be stable (i.e., have mean  = 0) with better confidence, which means some smaller prior variance. Within the method implemented in the MCMC.qpcr package, the default setting of the stability parameter for designated control genes allows them to vary 1.2-fold on average across experimental conditions, but this value can be increased to relax the stability assumption, or decreased down to 1, which would mean that the control genes are expected to be perfectly stable. Importantly, it is also possible to run the analysis in the “naive” form, without specifying any control genes.

In addition to restricting the mean change of the control genes in response to fixed factors, we might also wish to restrict their variances due to gene-specific random effects, *a_jg_* and *s_kg_* in the formula (2). MCMC.qpcr package provides an option to fix the variance components for the control genes at some specified value. For variance components of all non-control genes, flat non-informative priors are used by default, resulting in estimates approximating the maximum likelihood method [29], with an option to substitute them for two types of inverse Wishart priors. We found that, at least in the experiments described here, the inferred effect sizes and credible intervals were virtually unaffected by prior variance specifications and therefore chose not to discuss these options here, even though they may come useful in the future in the experiments specifically designed to quantify variances (such as, for example, in quantitative genetics).

### Model estimates and credible intervals

One notable advantage of the MCMC-based approach is that point estimates and credible intervals for any modeled effects can be easily calculated based on the parameter values sampled by the Markov chain. A credible interval is a Bayesian analogue of the confidence interval in frequentist statistics, and is defined as an interval that, with a specified posterior probability (e.g. 0.95), contains the true value of the parameter. Pairwise differences between conditions characterized by various factor combinations can also be computed, along with their credible intervals. This is useful for situations when factors have more than two levels. In addition to the fixed effects, credible intervals of variance components can be similarly examined; however, it must be remembered that interval estimates for variance components are robust only for data sets with many replications at the corresponding level of the model hierarchy.

### Testing for statistical significance

The question of interest in qPCR analysis is whether a specific treatment had an effect on a specific gene. The posterior distribution for *B_ig_* provides a direct answer to questions about probable effect size – or example, via a 95% posterior credible interval for each *B_ig_* term. Since within a single qRT-PCR experiment multiple comparisons of this kind are typically performed, there is a need to correct these results for multiple testing (but see [31] for a discussion of the multiplicity issue in this context). There are also many strategies for full Bayesian model selection that naturally account for multiple testing, e.g. [32]. But these are computationally intensive, and cannot be straightforwardly applied in non-Gaussian settings such as ours.

Although it is less natural to do so under the Bayesian paradigm, one may also use posterior tail areas to construct a procedure that behaves very much like a classical significance test. Specifically, define the two-sided Bayes tail area *p_ig_* as twice the fraction of all sampled parameter values for *B_ig_* that cross zero with respect to the posterior mean. These values would correspond to *p_MCMC_* calculated within the MCMCglmm package [29]. We treat the *p_ig_*'s as if they were classical p-values and correct them for multiple testing using a method that controls the false-discovery rate (FDR).

For large MCMC samples, our definition of *p_ig_* based on posterior tail areas is usually sufficient. But the lowest non-zero p-value that can be thus obtained is 2/M, where M is the size of the MCMC sample. To derive lower p-values based on a limited MCMC run, we approximate the posterior distribution of each parameter by a normal distribution and calculate a Bayesian z-score (the mean of the posterior divided by its standard deviation). This yields a two-tailed p-value based on a standard z-test.

There are at least two reasons why proceeding in this manner yields a sensible, though not exact, test. First, the Bernstein–von Mises theorem implies that, under quite general conditions, the joint posterior distribution behaves asymptotically like a multivariate normal distribution centered at the maximum-likelihood estimate, and with inverse covariance matrix given by the Fisher information matrix [33]. This implies that Bayesian tail areas are asymptotically equivalent to classical p-values (see, e.g. [34]).

While this asymptotic guarantee may be cold comfort for researchers with modest sample sizes, it should be emphasized that even purely classical analyses of generalized linear mixed models yield significance tests that are valid only asymptotically (e.g. [35] pg 385). Indeed, the construction of exact significance tests that properly account for the sampling distribution of both random effects and variance components in mixed models is notoriously challenging, and an open area of statistical research.

Second, the key feature of a p-value is that it has a uniform distribution under the null hypothesis. We conducted a simulation study to check whether this fact holds for *p_ig_* calculated on the basis of the Bayesian z-score in a realistic scenario. The results were encouraging (see below), seemingly justifying the use of *p_ig_* as a classical test statistic.

### Conversion of qRT-PCR data to counts

The central procedure in our method is the transformation of raw Cq values into molecule counts. In principle, this can be achieved using absolute quantification curves [11], which would need to be constructed for all genes. One disadvantage of this approach is that generation of such calibration curves must rely on an independent method of molecule quantification, which ideally should be more precise than qPCR. Here we explored an alternative approach, which relies on the knowledge of amplification efficiency (*E*, the factor of amplification per cycle) and the *Cq* of a single target molecule (*Cq*1). The counts can then be obtained using the formula: 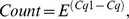



rounded to integer. (3)

**Figure 1:**
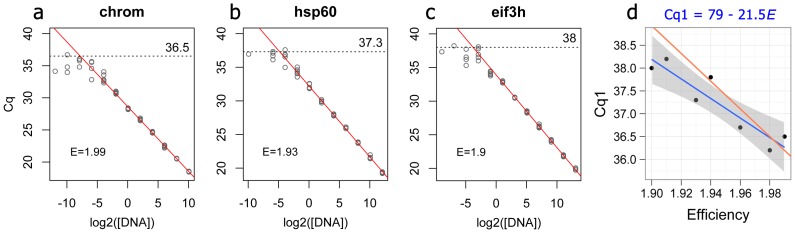
Effect of target concentration on *Cq* variance and estimation of the *Cq* for a single target molecule (*Cq*1). (a–c) Examples of amplification of different gene targets from a series of four-fold template dilutions with six-fold technical replication. The red line is the linear regression across 6–7 most concentrated dilutions where the increase in variance was not yet pronounced. The amplification efficiency (*E*, the factor of amplification per cycle), calculated from the slope of this regression [4], is listed on each panel in the lower left corner. The dotted horizontal line marks the visually determined *Cq*1. (d) Estimated *Cq*1 values for all tested targets plotted against their amplification efficiency. The blue line is linear regression, the shaded area corresponds to 95% confidence interval. The red line is the correlation expected from the efficiency-based PCR model [4] and *Cq*1 = 36 for a gene with *E* = 2.

To directly estimate *Cq*1 for several gene targets and evaluate the extent of its variation, we amplified seven targets from a series of four-fold cDNA dilutions that extended into the range of less than one target molecule per amplification trial with six technical replicates per dilution (Fig. 1). As expected, for all targets the variance between technical replicates increased as the average *Cq* per dilution began to exceed 30 (Fig. 1 a–c), presumably because of more and more pronounced effect of shot noise due to lowering number of molecules sampled per trial. We assumed that higher *Cq* values in dilutions where not all of the six amplification trials were successful likely corresponded to single-molecule amplification events [26], and estimated *Cq*1 visually (dashed lines on Fig 1 a–c).

The *Cq*1 values were negatively correlated with the amplification efficiency estimated from 6–7 higher-concentration dilutions (blue line of Fig. 1 d, p = 0.005). This correlation was reasonably close to what is expected under the model with the single efficiency parameter [4] assuming *Cq*1 = 36 for a target with efficiency *E* = 2 (red line on Fig. 1 d), although the slope of the real regression was notably shallower. Below we show that the results of relative quantification within our method are relatively robust to mis-specification of the *Cq*1 value, so the empirical formula describing the regression on Fig. 1 d (*Cq*1 = 79–21.5 *E*) provides a reasonable approximation if the experimental estimates of gene-specific *Cq*1 values are not available. Moreover, as we describe in the subsequent section, the results were virtually identical even when we simply assumed the same *Cq*1 = 37 for all genes.

### Cq1 and goodness of fit

**Figure 2:**
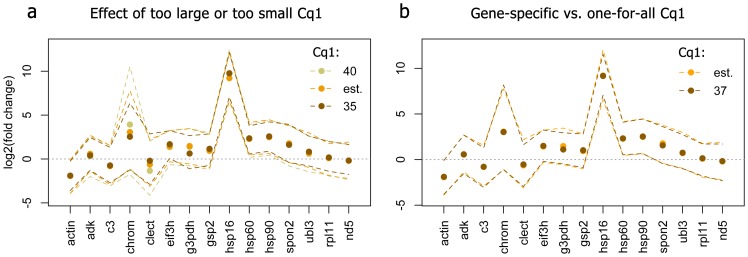
Effect of *Cq*1 setting on the point estimates and credible intervals of the fixed effects of stress in the coral dataset, according to the naive model. The points are posterior means, the 95% credible intervals are denoted as dashed lines connecting upper and lower interval limits across genes, to better visualize changes in their width. (a) Comparison of the results based on formula-approximated *Cq*1 (“est.”) with analyses assuming the same inflated (40) or diminished (35) *Cq*1 for all genes. The three most affected genes are *clect*, *chrom*, *g3pdh*, and *hsp16*, which were so low-abundant in at least one of the experimental conditions such that many of their qPCR trials were empty. (b) Comparison between analyses with formula-based *Cq*1 (“est.”) and uniform *Cq*1 = 37 for all genes.

**Figure 3:**
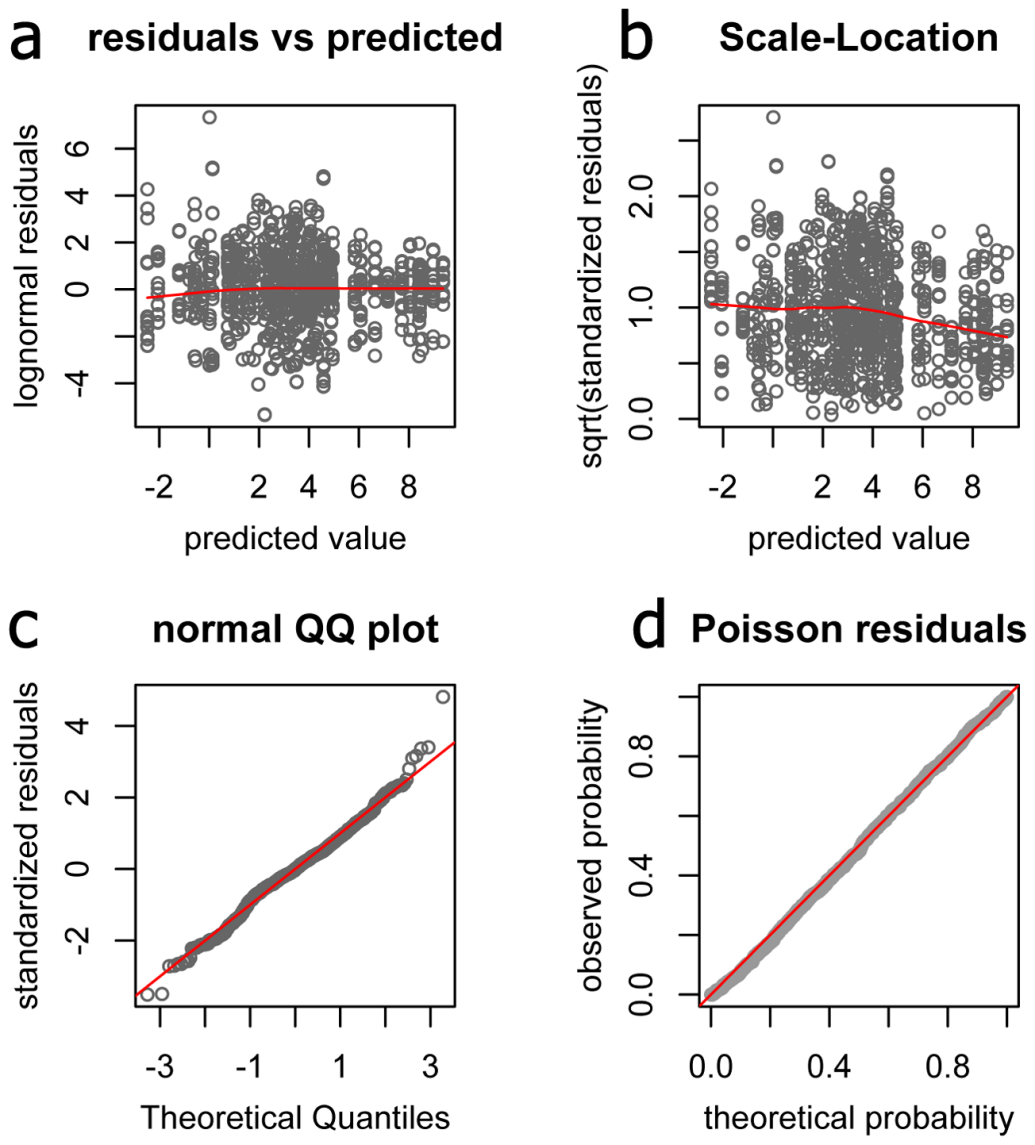
Goodness-of-fit characteristics of the naïve model applied to the coral stress dataset, for the *Cq*1 = 37 setting for all genes. (a) Plot of lognormal residuals against predicted values to test for linearity. (b) Scale-location plot to test for homoscedasticity. A good fit is corroborated by the lack of pronounced mean trend in these two plots (red lines). (c) Plot of quantiles of standardized lognormal residuals against theoretical quantiles of the normal distribution. Red diagonal corresponds to the exact match. (d) Probabilities of experimental Poisson residuals plotted against their theoretical probabilities for one of the MCMC samples. All MCMC samples show the same nearly perfect fit to the Poisson expectations.

The mis-specification of *Cq*1 can be expected to affect primarily low-abundant genes, which lie in the “shot noise zone” (*Cq* >30, Fig. 1 a–c) and for which the range of abundances might be bounded by zero (i.e., empty amplification trials in the data). Fig. 2 shows the results of a naïve model (no control genes specified) estimating the main effect of heat stress in our coral dataset, fitted to the data converted either with formula-approximated *Cq*1 (Fig. 1 d) or the same *Cq*1 for all genes, which was either too large (40), or too small (35). As expected, too large or too small *Cq*1 (Fig. 2 a) affected point-estimates considerably only for the least-abundant genes which were zero-bounded (*chrom*, *clect, g3pdh,* and *hsp16*). For these genes, larger *Cq*1 resulted in larger inferred fold-change and broader credible intervals, with the exception of *hsp16*. For other genes, the point-estimates were unaffected, while the credible intervals exhibited the same tendency to scale with *Cq*1, but to a much smaller extent than for the zero-bounded genes. Assuming a single *Cq*1 = 37 for all genes had virtually no effect on the inference, compared to the more accurate formula-approximated *Cq*1 data (Fig. 2 b). Goodness-of-fit characteristics were visually indistinguishable between the dataset converted using formula-approximated *Cq*1 and the dataset with the same *Cq*1 = 37 for all genes (Fig. 3). The departures from perfect linearity (Fig. 3 a) and trend towards higher variance at the low end of predicted values (Fig. 3 b) were negligible, and the distribution of lognormal residuals was very close to normal (Fig. 3 c). The probabilities of Poisson residuals [36] were nearly exactly as expected (Fig. 3 d), supporting the basic assumption that the higher variance at higher *Cq* is due to Poisson fluctuation in numbers of sampled molecules [28].

### Effect of control genes and fixation parameters

**Figure 4:**
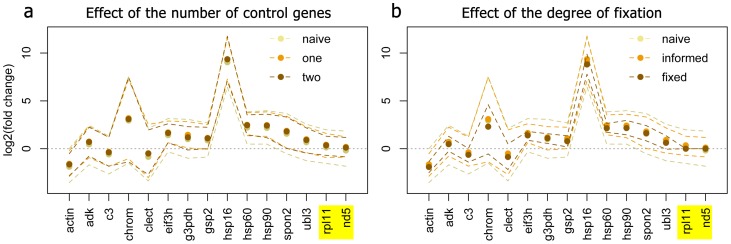
Effect of adding control genes (highlighted) as priors on the estimates of fixed effects of stress in the coral dataset. The points are posterior means, the 95% credible intervals are denoted as dashed lines connecting upper and lower interval limits across genes. (a) Comparison between naïve model (no control genes specified) and two informed models, with one (*nd5*) or two (*nd5, rpl11*) control genes specified (the control genes were allowed to change 1.2-fold on average in response to fixed factors). (b) Comparison of the naïve and two-gene informed model to the two-gene fixed model, in which the same control genes were required to be absolutely stable.

Figure 4 a shows the comparison between naïve model and two informed models in which either one (*nd5*) or two (*nd5* and *rpl11*) control genes were specified but allowed to change 1.2-fold on average in response to the fixed factors. Inclusion of control genes did not have much effect on the point-estimates, but led to considerable narrowing of the credible intervals. The most narrowing was seen after adding one control gene; addition of the second one had much less effect. Figure 4 b compares the results of the naïve and informed model with two control genes (the same as on Fig. 4 a) with the “fixed” model in which the two control genes were required to remain perfectly stable. Complete fixation of control genes results in very narrow credible intervals; however, perfect stability of expression of any one gene is considered to be an unrealistic assumption [15], and hence the power gained in the fixed model may be coming at the expense of accuracy.

### Analysis of smaller datasets

**Figure 5:**
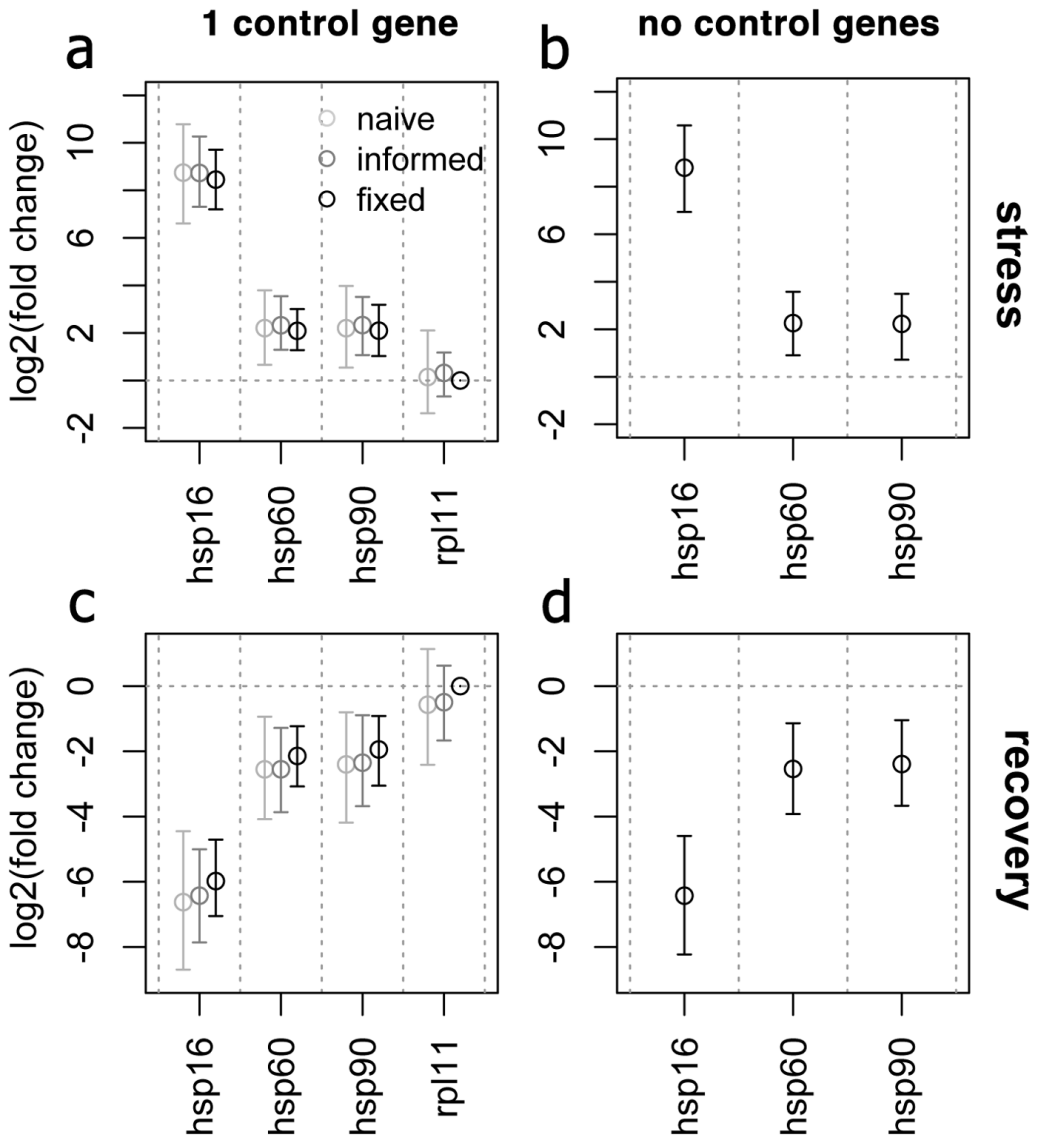
Analysis of small unbalanced subsets of the coral stress data. (a, c): Three heat shock protein genes plus one control gene (*rpl11*), analyzed using naïve, informed, and fixed model. (b,d): Analysis of only the three heat-shock proteins. (a,b) – effects of stress; (c,d) – effects of recovery. The points are posterior means, the whiskers denote 95% credible intervals. Bayesian analysis infers the same fold-changes regardless of specification of the control gene and even in its absence.

Good performance of the naïve model, not relying on any control gene information, may not be too surprising for a dataset that contains many genes demonstrating various expression patterns, but will it work when genes are few and their expression patterns are unbalanced? To explore this issue, a smaller dataset was extracted from the coral stress data containing only four genes: a control gene *rpl11* and three most highly regulated genes, the heat shock proteins *hsp16*, *hsp60* and *hsp90* (Fig. 5 a, c). Analysis of this small dataset recovered exactly the same regulation patterns for the four genes as observed in the whole dataset, irrespective of whether the control gene was specified as a prior or not (Fig. 5 a, c). Moreover, even when the control gene was removed from the dataset, leaving only the three genes that were strongly up- or down-regulated in concert, the same results were recovered (Fig. 5 b, d).

### The “classic” model

Some datasets may not conform to the assumption of our model (2) that the variation in template loading between samples, *t_k_*, can be modeled as a single Gaussian distribution across all experimental conditions. If for some conditions the RNA samples systematically show substantially lower concentration and/or quality, the model will infer down-regulation of all genes under these conditions. Accounting for such a bias would unavoidably require reliance on control genes for normalization. We therefore implemented our single-model MCMC-based approach involving the multi-gene normalization procedure [16]. The normalized data are analyzed using the same model as given by formula (2) only lacking the *t_k_* term since this variation is supposed to be subtracted out. Since normalization procedure would preclude the use of counts as the response variable, we have to fall back to log-transformed expression values and run a lognormal rather than Poisson-lognormal model. The data for this analysis is prepared by converting raw *Cq* values into natural logarithms of relative abundances (*Ra*) while correcting for the efficiency of amplification using the transformation introduced previously [3], [23]:
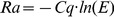
(4)


We call this model “classic” since it is based on earlier developments [22]–[24] and lacks the main advancements proposed in this paper, such as the use of generalized linear modeling to account for higher variance of low-abundant targets and the possibility to analyze the data without reliance upon control genes. The one innovation this model offers, however, is a single-model rather than gene-by-gene analysis, which is expected to boost the power considerably since the model draws evidence from all genes simultaneously [25].

**Figure 6:**
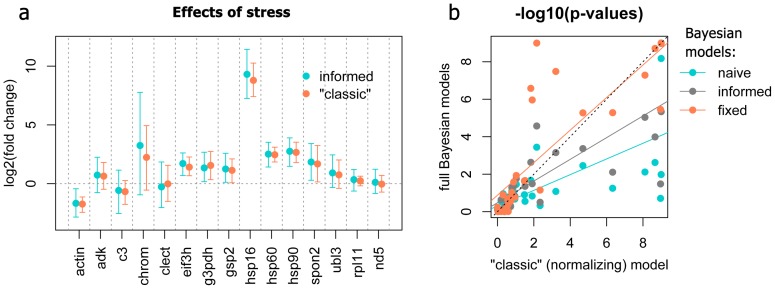
Comparison of the procedure based on multigene normalization followed by single-model MCMC analysis (“classic” model) to full Bayesian models. (a) Point-estimates and credible intervals for the effects of stress in coral dataset inferred by the informed model (allowing control genes *rpl11* and *nd5* to change 1.2-fold on average) and “classic” model based on normalization using the same two control genes. Points are posterior means, the whiskers denote 95% credible intervals. (b) Comparison of the power of the “classic” model (transformed p-values along the horizontal axis) to the power of full Bayesian models (see legend). The colored lines are linear regressions to illustrate trends (no statistical implications intended), the black dotted line is 1∶1 correspondence. “Classic” model generates the the credible intervals that are as narrow as under the fixed model, but relies on more realistic assumptions.

For the coral stress dataset, the “classic” model generated virtually identical point-estimates of fold changes as the full Bayesian models (Fig. 6 a), while being comparable in the width of the credible intervals to the most powerful model of the three, the fixed model (Fig. 6 b).

### Bayesian p-values

**Figure 7:**
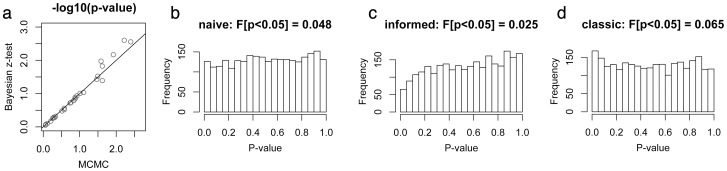
Properties of p-values based on Bayesian z-test. (a) Correspondence between p-values based on posterior tail areas (horizontal axis) and z-test based p-values (vertical axis) for the stress and recovery effects in the coral dataset. (b–d) Frequency distribution of z-test based p-values obtained using naïve (b), informed (c) and “classic” (d) models from datasets simulated under null hypothesis. The fraction of simulated p-values that are less than 0.05 is given above each plot.

The p-values based on Bayesian z-test agreed well with *p_ig_* calculated directly from the posterior tail areas (Fig. 7 a), supporting the validity of the z-test approach. To explore the distribution of z-test derived p-values under null hypothesis under three models (naïve, informed, and “classic”) we generated three null datasets based on our coral data by subtracting the main effects inferred by each of the models from the efficiency-corrected *Cq* values (*Cq* ^corrected^  =  *Cq* · log_2_(*E*), the “perfect world” *Cq* values that would have been observed if all genes were amplified with the efficiency of exactly 2, [23]). We then ran 100 re-analyses of these datasets while randomly shuffling samples between experimental conditions and calculated Bayesian p-values for the main effects and their interaction for each gene, resulting in 2,600 p-values per model. The frequency distribution of these p-values was nearly ideally uniform under the naïve model (Fig. 7 b) and only slightly skewed under informed and “classic” models (Fig. 7 c,d). This result suggests that the test based on Bayesian p-values will be of correct size for the naïve model, will be slightly more conservative than the nominal alpha value under the informed model, while under “classic” model it might be very slightly less conservative. These deviations of Bayesian p-values from the uniform distribution under the null hypothesis are probably negligible for all practical intents and purposes. In particular, they should not preclude the applicability of multiple testing correction controlling for false discovery rate [37].

### Comparison with other methods

**Figure 8:**
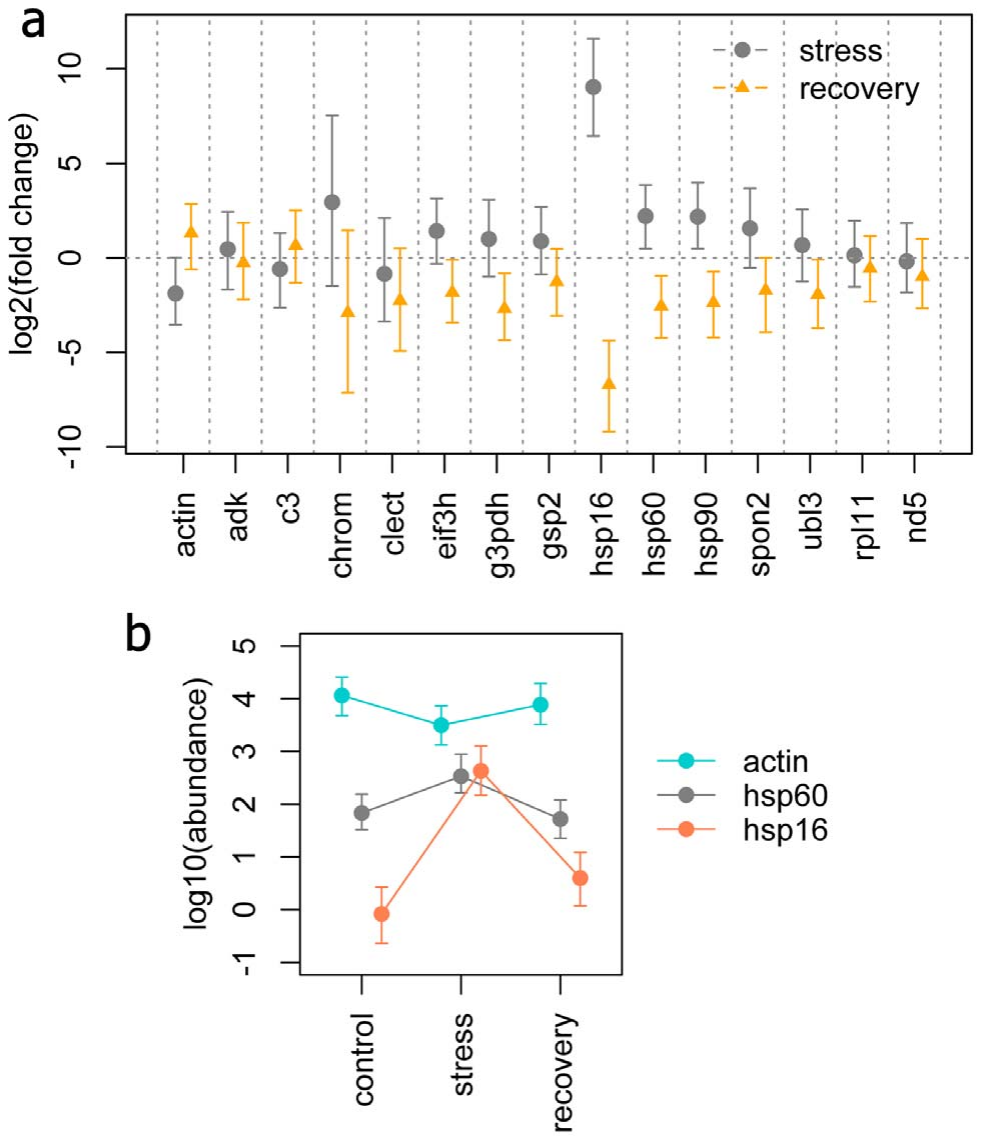
Visualization of the results of Bayesian analysis of the coral stress dataset. (a) Effects of stress and recovery under naïve model. It can be seen that recovery gene regulation is basically a mirror image of stress response. (b) Transcript abundances of selected genes (see legend) across conditions of interest. The points are posterior means, the whiskers denote 95% credible intervals.

**Figure 9:**
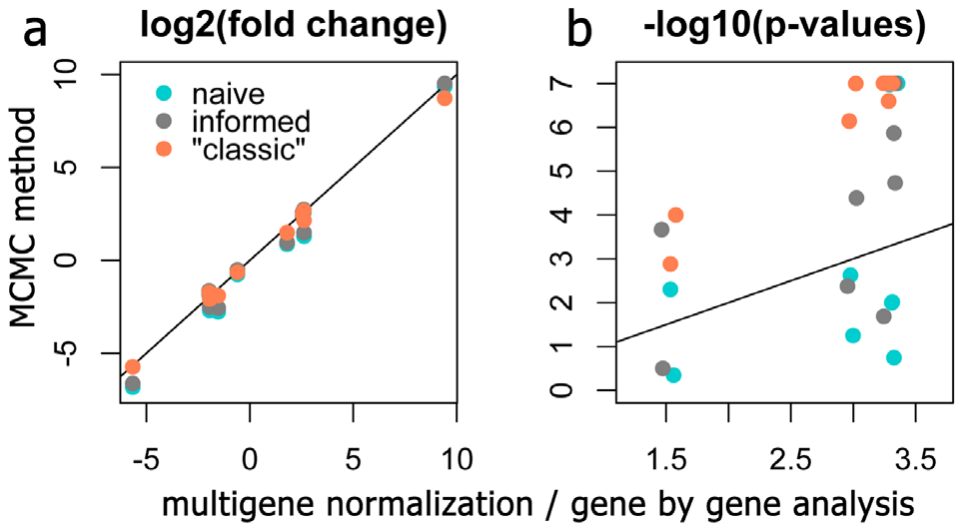
Comparison of the Bayesian analysis of the coral stress dataset to the previously published results based on multigene normalization and gene-by-gene linear mixed modeling [23]. (a) The match between the fold-changes inferred in the current reanalysis and previously reported changes, for naïve, informed and “classic” models. (b) Correspondence between p-values under the naïve, informed, and “classic” models (see legend on panel a) and the previously reported p-values. Points above the line indicate higher power (lower p-values) of the new models. The reanalysis p-values were derived by the Bayesian z-test.

Figure 8 presents the results of MCMC.glmm analysis of the coral stress dataset using the naïve model, this time showing both the effect of stress and subsequent recovery, which were the focus of the original analysis based on multi-gene normalization and gene-by-gene linear mixed modeling [23]. Notably, the naïve model was able to recapitulate the previous findings without making any assumptions regarding control genes. As before, the main effects of stress included very strong up-regulation of *hsp16* (a small heat-shock protein), more modest up-regulation of large heat shock proteins *hsp60* (Fig. 8 c) and *hsp90*, and down-regulation of *actin* (Fig. 8 b); during recovery this pattern was reversed. For these four response genes reported the original paper [23], we plotted their inferred fold-changes during stress and recovery under naïve, informed and “classic” models against the originally reported values (Fig. 9 a), observing a very close match. Plotting the new *p*-values (based on Bayesian z-test) against the previous results (Fig. 9 b) illustrated that the naïve model is less powerful (most points are below the diagonal), informed model appears about as powerful, and “classic” model is considerably more powerful than the original analysis. The new models, including the naïve one, suggested additional significantly changing genes (such as down-regulation of glyceraldehyde-3-phosphate dehydrogenase, *g3pdh*, during recovery, Fig 8 a), which were not detected previously and not included in the plots on Fig. 9. The much higher power of the “classic” model compared to the previous gene-by-gene analysis confirmed the expectation that a single-model approach, drawing evidence from all genes at once, would be more powerful even if it is based on the same data pre-processing pipeline (log-transformation and multigene normalization).

**Figure 10:**
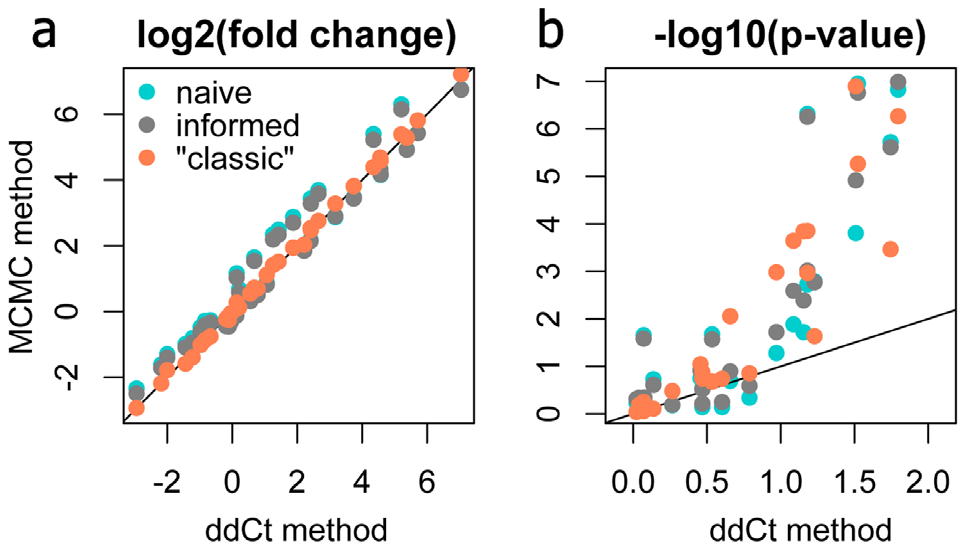
Comparison of the Bayesian method to the delta-delta-Ct method, reanalyzing data from [38]. (a) Fold-changes inferred by the naïve, informed and “classic” models plotted against fold-changes derived by the delta-delta-Ct analysis with a single control gene. (b) Comparison of p-values derived by the Bayesian z-test from naïve, informed, and “classic” models (see legend on panel a) with p-values obtained by pairwise t-tests within the delta-delta-Ct pipeline. On both panels, the line denotes 1∶1 correspondence. On panel b, points above the line indicate higher power (lower p-values) of the new models.

To see how the new methods compares to the classic delta-delta-Ct procedure [2], we re-analyzed the data comprising Figure 6 in [38]. This experiment examined the effect of a single factor, treatment, with five levels (control and four different heat stress regimes) on the expression of nine response genes in cultured murine fibroblasts. A single control gene (glyceraldehyde-3-phosphate dehydrogenase, *g3pdh*) was used to calculate fold-changes relative to the control condition while correcting for amplification efficiencies [4]. The dataset included three biological replicates per factor level and three technical replicates per RNA sample. For naïve and informed models, we converted the original *Ct* data to counts assuming *Cq*1 = 37 for all genes. The “classic” model recapitulated the delta-delta *Ct* results nearly exactly, as expected, but it is notable that very similar fold-changes were also inferred by the naïve model, which did not use any control gene information (Fig. 10 a). Moreover, the power of all our models (even the naïve one) was substantially higher than the power of the original analysis based on pairwise t-tests (Fig. 10 b).

## Discussion

### qRT-PCR data as counts

The purpose of *Cq*-to-counts transformation described here is to create a dataset in which the increase in variance at the low gene expression values (Fig. 1 a–c) can be adequately accounted for by the relative quantification model. General reasoning, earlier literature [28], and our analysis presented here (Figure 3 d) suggest that this increase in variance is likely due to Poisson-distributed fluctuations when the number of sampled molecules is small. It has been previously shown that the amplification products obtained from DNA samples so highly-diluted that only a fraction of PCR trials are successful indeed correspond to amplification of individual molecules [26]. This substantiates our approach of determining *Cq*1, the number of PCR cycles required to amplify a single target molecule, through analysis of over-extended dilution series (Fig. 1 a–c). It is notable, however, that the *Cq*1 values thus determined show less pronounced correlation with the efficiency of amplification (Fig. 1 d) than expected under the simple qPCR model with a single efficiency parameter [4]. We must therefore caution that even though *Cq*-to-counts data transformation achieves its primary purpose, enabling quantification of very low-abundant targets within the general relative quantification framework, further qPCR model development might be required to ensure accurate absolute quantification of individual molecules.

### The Poisson-lognormal Bayesian model

This model is designed to be universally applicable in qRT-PCR. It properly handles the full range of target abundances down to the individual molecule level, derives information from no-amplification trials, and provides a possibility to specify control genes with the desired degree of confidence. In the same time, it is fully flexible in terms of experimental designs that it can accommodate, and would handle any number of fixed and random effects and their interactions.

### The “classic” and lognormal Bayesian models

The “classic” model represents a viable alternative to the full Bayesian analysis especially for the cases when the quantity and/or quality of the RNA samples varies systematically across experimental conditions, and when the expression levels are not too low (i.e., the majority of Cq values are below 30). The model is considerably more powerful than previously used procedures based on the same principles of data processing (Fig. 9 and 10), highlighting the advantage of joint analysis of expression of all genes within the same model. It is also more powerful that any of the full-Bayesian models described here, with the exception of the fixed model (Fig. 7). The power is expected to improve with more genes in the dataset. Although the power comes at a cost of strong assumptions concerning control gene stability, the “classic” model only requires their average expression to be constant, which is considered acceptable in the qRT-PCR analysis field [16]. Compared to the fixed model, which requires absolute stability of each of the control genes, this is objectively a more realistic assumption.

When the targets' abundances remain relatively high and there is little concern about shot noise, it is possible avoid making assumptions concerning *Cq*1 values but still use the full Bayesian approach. To do this, the MCMC.qpcr package implements the model (2) in a lognormal form, based on log-transformed efficiency-corrected data (4). In this implementation, full range of possibilities is retained for specifying (or not) any control genes as priors.

### Choice of model: power versus risk of bias

The models described here might be ranked according to their increasing power, manifested as narrowing credible intervals, in the order naïve – informed – ”classic”/fixed. It is encouraging that, at least for the two qRT-PCR datasets that we examined here, all models gave very similar point-estimates of gene expression changes when applied to the same data (Fig. 9 and 10), indicating the lack of major biases induced by the increasing number and strength of assumptions within the models. Still, it is important to remember that higher power brings about the risk of biased inference, especially since the power comes as a result on increasingly stronger assumptions. The fixed model, for example, assumes complete stability of the control genes, which is not realistic in gene expression studies and therefore should be avoided in qRT-PCR. On another hand, although the naïve model might seem preferable since it makes the least assumptions, it underutilizes the possibilities provided by the Bayesian framework, which is rather undesirable unless the analysis has to be kept strongly conservative. We feel that for the majority of day-to-day qRT-PCR cases the best choice balancing the conservatism and good use of available information would be the informed model with one or two control genes, or the “classic” model with three-four control genes.

### Control genes

From a practical qRT-PCR standpoint, arguably the most attractive feature of the full Bayesian methodology is its robustness with respect to the number and even the very presence of control genes among the analyzed targets. Even when control genes are not specified as priors (naïve model), the model still can successfully discount the variation due to different amounts of template across samples (Fig. 5 and 6). This “self-normalizing” capacity highlights the advantage of whole-dataset mixed model analysis. The model regards sample-specific effects as a random variable drawn from the same normal distribution, which helps disentangle them from the regulation patterns due to fixed effects. The accuracy of this inference benefits more from the larger number of samples than from the number of genes or technical replicates, since the parameters of the distribution underlying sample-specific variation can be estimated with better confidence with more samples.

Even when naïve model analysis is intended, it would be prudent to keep one or two presumably stable genes among analyzed targets: such genes could serve as indicators that the model performs reasonably, in addition to diagnostic plots (Fig. 3). The stability of the control genes can be validated by running a naïve model, and it is also possible to use the naïve model to identify potential control genes in the first place. It must be emphasized, however, that inferring control genes from an experimental dataset and then using them to analyze the same dataset may lead to biased inference due to circularity. Ideally, control genes should be selected based on an independent experiment or other supporting data. If there is no such prior information, at the very least an alternative method of control gene selection should be used, such as non-parametric geNorm [16]. A single control gene seems to be sufficient to gain good power within the informed model (Fig. 5 a).

Even though our fixed model makes assumptions that are hardly ever realistic in analysis of gene expression [15], absolute fixation of a control target could be useful in certain experimental designs. One example is quantifying relative amounts of DNA targets, such as the amount of parasite or pathogen DNA relative to the DNA of the host. In this case, the absolute fixation of the control (host-specific) gene would generate the required relative quantification results, with the most power (Fig. 5 b and Fig 7).

### Implementation

The MCMC.qpcr package accompanying this paper (File S1) comprises the complete pipeline of qRT-PCR analysis based on the methodologies described here. The complexity of the model (2) remains mostly hidden from the user since the function that builds the model formula, constructs priors, and runs MCMC chain only requires the user to specify the fixed and random effects (if any). The functions to extract the inferred gene expression changes, to calculate their credible intervals and statistical significance, and to visualize the results (such as on Fig. 5, 6 and 8) also require relatively simple and intuitive user input. The tutorial accompanying the MCMC.qpcr package (File S2) explains the whole process step by step, and we believe that this will make our methodologies accessible even to beginner R users.

## Methods

The qPCR analysis of over-extended dilution series (Fig. 1) was performed on a Roche LightCycler 480 instrument equipped with a 384-well block, using LightCycler 480 SYBR Green I Master kit (Roche). The primer sequences, composition of the reaction mix, cycling parameters and procedures for RNA isolation and cDNA synthesis were the same as described previously [23]. All the analyses and development of data processing functions were accomplished in R software environment [39].

## Supporting Information

File S1 **MCMC.qpcr R source package.**
(TAR.GZ)

File S2 **Tutorial for the Bayesian qRT-PCR analysis methodology as implemented in the MCMC.qpcr package.**
(PDF)
