# Supplementary material for: No Control Genes Required: Bayesian Analysis of qRT-PCR Data
Source: PLoS One. 2013 Aug 19;8(8):e71448. doi: 10.1371/journal.pone.0071448 (PMC3747227; doi:10.1371/journal.pone.0071448)
Supplement: File S2 — Tutorial for the Bayesian qRT-PCR analysis methodology as implemented in the MCMC.qpcr package. (PDF) [file pone.0071448.s002.pdf]

Bayesian analysis of qRT-PCR data using MCMC.qpcr package: a tutorial

Mikhail V. Matz
University of Texas at Austin
matz@utexas.edu

July 2013

download MCM.qpcr package (source file) at CRAN, or here:
http://www.bio.utexas.edu/research/matz_lab/matzlab/Methods_files/MCMC.qpcr_1.0.tar.gz
(Please make sure the file does not get un-zipped automatically after download!)

Coral stress-recovery experiment

The exercises in this tutorial are based on the dataset '`coral.stress`', provided with the package. The experiment that generated this dataset addressed the effects of heat-light stress and subsequent recovery on gene expression in a reef-building coral *Porites astreoides* (Kenkel et al. 2011). Briefly, eight individual coral colonies were fragmented into 4 pieces each and allowed to acclimatize in common benign conditions for four days. The next morning, two fragments of each colony were placed into a stressful environment (elevated heat and light). At midday on the same day, when the stress intensity was the highest, one stressed fragment and one control fragment (remaining in the benign conditions) were sampled, representing the first sampling timepoint. In the end of the same day, the second stressed fragment was put back into the benign environment for recovery. This fragment, along with the remaining control fragment, was sampled at midday on the following day (the second timepoint, corresponding to recovery). Expression of 15 genes, 5 of which were putative control genes, was assayed by qRT-PCR on Roche LightCycler 480 instrument with SYBR detection. In the original paper (Kenkel et al. 2011), the analysis included correcting for amplification efficiencies, normalization by the 3 genes that proved to me most stable according to geNorm test (Vandesompele et al. 2002), followed by linear mixed model analysis on a gene-by-gene basis (Poletto et al. 2006).

This dataset is interesting from the analytical standpoint because of three reasons. First, one of the main effects of interest is the interaction term, Condition:Timepoint, describing the gene regulation in coral fragments that were first stressed and then allowed to recover. Evaluation of the interaction term necessitates the use of linear models or ANOVA rather than non-parametric methods (Steibel et al. 2009). Second, the dataset includes an important random effect, the identity of the individual coral colony, potentially affecting the baseline level of expression of some genes, which prompts the use of a linear mixed model rather than a simple linear model. Finally, several genes were so down-regulated that they became undetectable by qRT-PCR in a considerable number of trials, precluding a straightforward use of log-transformation typical of qRT-PCR analysis (Vandesompele et al. 2002; Luu-The et al. 2005).

Cq data format

The main input is the table of raw Cq ("cycle of quantification" values, which may be called 'Ct' depending on the kind of qPCR instrument) containing columns of Cq values (one column per gene) and columns of conditions (fixed and random factors). Gene and condition names should be given as column titles. In addition to normal Cq values, the Cq columns may contain entries '`NA`' (missing data, which might be entered because the observed amplification product did not have the expected melting peak) and '`-1`' (no amplification - presumably because there was not a single target molecule in the analyzed

aliquot). Technical replicates should not be averaged but instead listed as separate rows. To view the format, use `head()` to print out the first ten lines of the '`coral.stress`' dataset:

```
library(MCMC.qpcr)
data(coral.stress)
head(coral.stress)
     sample individual condition timepoint hsp16 actin   adk    c3 chrom clect eif3h
   1      1         s1   control       one 36.62 22.44 31.54 29.57    NA 33.93 31.69
   2      1         s1   control       one -1.00 22.42 31.24 28.83 37.76 32.99 32.02
   3      2         s1      heat       one 26.89 23.27 30.92 27.98 30.70 34.74 29.65
   4      2         s1      heat       one 27.08 23.34 31.71 28.06 31.28 33.95 29.73
   5      3         s2   control       one    NA 23.24 33.22 27.08 34.95    NA 31.41
   6      3         s2   control       one -1.00 23.94 33.13 27.73 34.70 -1.00 32.90
```

Here, the first four columns denote random and fixed factors. Rows with exact same entries in these four columns are technical replicates (two per sample in this case). It is OK to have occasional dropouts of technical replicates, as long as the dropout rate is not correlated with any of the factors.

One critical random factor for the analysis is '`sample`', denoting individual cDNA preparations. This factor will be used to infer different template loadings in qPCR reactions, which serves as a functional equivalent of normalization in qRT-PCR. The '`sample`' column must be present in the table, does not matter in which position.

Other random factors would typically be grouping factors, such as block, plot, tank, cage, genotype, etc. In this case, the '`individual`' random effect denotes the coral colony that was fragmented into clonal pieces that were subject to treatments.

Finally, the experimental design includes two fixed effects, '`condition`' ('`control`' or '`heat` ') and '`timepoint` ' ('`one`' corresponding to the day of stress, '`two`' corresponding to the following day when the corals were allowed to recover).

Such a table can be assembled in spreadsheet editor software, such as MS Excel, from raw Cq data exported by the qPCR instrument. While naming the factors and genes, remember that in R the column names cannot start with a number and cannot contain spaces (or rather, spaces will be converted to full stops, '.'). Save the table as "comma-delimited values" (with the extension .csv), and use R function `read.csv()` to import it into R:

```
mydata=read.csv("name-of-my-file.csv")
head(mydata)        # to see if the dataset was imported correctly
```

Amplification efficiencies

The second input is a two-column table of qPCR efficiencies for each gene. The efficiency (*E*) is the amplification factor per PCR cycle, and is needed for proper Cq to counts conversion. To estimate *E*, we recommend the dilution series method (Pfaffl 2001), where a series of 8-10 2-fold template dilutions is analyzed by qPCR. The template should be a previously amplified fragment, pre-diluted 100,000-fold to make Cq values fall within the realistic range. The package contains a dataset of dilution series results called '`dilutions`' and a function `PrimEff()` that would calculate amplification efficiencies, plot the regressions (Fig. 1) and summarize the results in a form of a table:

```
data(dilutions)
dilutions
             dna    cq  gene
   1  1.000000000 19.80 eif3h
   2  1.000000000 19.75 eif3h
   3  1.000000000 19.99 eif3h
   4  1.000000000 19.99 eif3h
   5  1.000000000 19.78 eif3h
   6  1.000000000 20.06 eif3h
```

```
   7  0.250000000 21.73 eif3h
   8  0.250000000 21.88 eif3h
   9  0.250000000 21.82 eif3h
   10 0.250000000 21.78 eif3h
   11 0.250000000 22.01 eif3h
   12 0.250000000 21.80 eif3h
   13 0.062500000 24.10 eif3h
   14 0.062500000 23.92 eif3h
   15 0.062500000 24.18 eif3h
   16 0.062500000 24.27 eif3h
   ...
```

```
PrimEff(dilutions) # Fig. 1
      gene     E E.minus.sd E.plus.sd intercept
   1 chrom 2.017       2.00      2.03      18.6
   2 eif3h 1.900       1.89      1.91      19.8
```

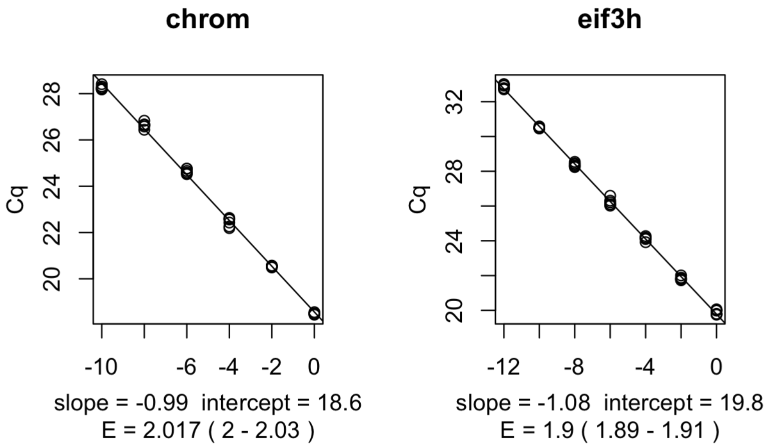

Figure 1. `PrimEff()` plots of regression slopes and PCR efficiencies based on dilution series.

The input table for this analysis can once again be created in Excel, and should have the same order of the columns as the '`dilutions`' example: first is RNA concentration (which may be arbitrary, 1/[dilution factor], as it is here), second is Cq (or Ct), third is gene name. Names of the columns do not matter as long as their order is the same. The input may contain concatenated data for several genes.

When all the gene targets are analyzed for qPCR efficiency, the results should be put together in a table that will be used by MCMC.qpcr analysis:

```
data(amp.eff)
amp.eff
       gene efficiency  Cq1
   1  hsp16       1.90   NA
   2  actin       1.95 36.5
   3    adk       1.99 36.8
   4     c3       1.99   NA
   5  chrom       1.98 36.5
   6  eif3h       1.90 38.0
   7  g3pdh       1.85 39.2
   8   gsp2       1.96 36.7
   9  hsp60       1.93 37.3
   10 hsp90       1.91 38.2
   11   nd5       1.97 36.2
```

```
12 rpl11       1.95 37.8
13 spon2       2.00 36.5
14  ubl3       2.00   NA
15 tgoln       2.00   NA
16  r18s       1.93   NA
17 clect       2.00   NA
```

There are two obligatory columns: gene name should appear in the first column, while *E* should be in the second column (the column names don't matter). The third column is optional and contains an experimentally estimated Cq of a single molecule of the target. The table should contain all the genes that feature in the main Cq table. It is perhaps needless to say that the gene names must be spelled exactly the same way in both tables (remember that R is case-sensitive).

Cq to counts conversion

This is the key transformation in the whole methodology, which makes it possible to apply generalized linear model to account for higher variation at the lower end of target abundances, and also to properly derive information from empty qPCR trials.

The conversion to approximate counts uses a simple formula:

$$\text{Count} = E^{\,(\text{Cq1} - \text{Cq})}\text{, rounded to integer ,} \qquad (1)$$

where *E* is the efficiency of amplification and Cq1 is the the number of qPCR cycles required to detect a single target molecule. Cq1 is the feature of the qPCR assay when run on a particular instrument, and its exact determination requires considerable effort. Fortunately, at least the 11 genes that we examined in detail turned out to have very similar Cq1, within 36-39 range, and we were able to demonstrate that the choice of Cq1 does not affect the results of relative quantification much as long as it is within this range. Assuming the same average-size Cq1= 37 for all genes resulted in good model fit and exactly the same results as the analysis with more accurately approximated Cq1. We therefore believe that Cq1=37 will work well for most tasks, as long as the goal is relative rather than absolute quantification. A more sophisticated way of specifying Cq1 is to approximate it based on the efficiency (*E*) using empirical formula Cq1 = 79 - 21.5*E*, but we cannot guarantee that the formula, which we developed for LightCycler 480, would be applicable to other instruments.

The function that performs the conversion is `cq2counts()`. In addition to the names of the main Cq dataset ('`data`' argument) and the name of amplification efficiencies dataset ('`effic`' argument), it also needs to be told which columns contain Cq values ('`genecols`' argument) and which columns correspond to conditions ('`condcols`' argument). The method of Cq1 setting is determined by '`Cq1`' parameter ("table" for experimentally estimated Cq1 listed as the third column in the efficiency table, "formula" for formula-based approximation, and any number to fix Cq1 for all genes at this value). We will specify `Cq1=37`:

```
data(coral.stress)
data(amp.eff)
dd=cq2counts(
      data=coral.stress,
      genecols=c(5:19),   # where the Cq data are in the data table
      condcols=c(1:4),    # which columns contain factors
      effic=amp.eff,
      Cq1=37
      )
head(dd)
      count  gene sample individual condition timepoint
   1      1 hsp16      1         s1   control       one
   2      0 hsp16      1         s1   control       one
   3    658 hsp16      2         s1      heat       one
```

```
4    582 hsp16      2          s1      heat       one
5     NA hsp16      3          s2   control       one
6      0 hsp16      3          s2   control       one
7    160 hsp16      4          s2      heat       one
8    142 hsp16      4          s2      heat       one
9      7 hsp16      5          s4   control       one
10     4 hsp16      5          s4   control       one
```

The resulting data frame (`dd`) has the raw Cq values efficiency-corrected and transformed into counts based on Cq1 = 37, also have all the counts "stacked" so they all are now in a single column.

The qRT-PCR model

Below I use the coral example to explain, in colloquial terms, how the qRT-PCR model is constructed. A reader inclined towards more statistically rigorous notation and language is encouraged to study our original paper introducing the method (Matz, Wright, and Scott, 2013). The model has a single response variable, the natural logarithm of transcript counting rate. The most basic explanatory variable in the model is 'gene', which accounts for different levels of expression between genes:

$$\ln(\text{rate}) \sim \text{gene}\ .$$

Such a primitive model, however, would be of little value since in qRT-PCR we are typically not interested in difference in expression between genes, but want to learn how the expression of each gene varies depending on the experimental treatments. To find this out, we augment our model with a series of terms describing gene-specific effects of experimental treatments. In our coral experiment, we have two treatments (or, using linear modeling terminology, factors): Condition with levels "control" and "heat" and Timepoint with levels "one" and "two", plus their interaction (i.e., we suspect that there might be some Timepoint-specific effects of Condition). This experimental design is incorporated into the model as follows:

$$\ln(\text{rate}) \sim \text{gene} + \text{gene:Condition} + \text{gene:Timepoint} + \text{gene:Timepoint:Condition}\ ,$$

where the colon indicates interaction, essentially standing for "-specific effect of ". The model is fully flexible, not being limited to a particular number of factors, number of levels within each factor, or presence-absence of interactions.

Even though our model specification now seems to contain all the terms we want to estimate, we must take care of other important sources of variation that, while being of no real interest to us, must be taken into account to ensure that the model is accurate and powerful. The most important of these is the random effect of the biological replicate (i.e., an individual RNA sample), accounting for the variation in quality and/or quantity of biological material among samples. The designation "random effect" implies that we are not interested in actual estimates of each sample's quality or quantity, but simply want to partition out the corresponding variance. Random effects are imagined as random variables drawn from an underlying distribution the variance of which the model will estimate. In the simplified notation that we use throughout this section, we will italicize the names of random factors, to discriminate them from the factors of primary interest ("fixed factors") that we discussed before:

$$\ln(\text{rate}) \sim \text{gene} + \text{gene:Condition} + \text{gene:Timepoint} + \text{gene:Timepoint:Condition} + \textit{sample}\ .$$

Note that, since the variation in cDNA quality and/or quantity affects all genes in a sample in the same way, this random factor is not gene-specific. The introduction of this random factor into the qRT-PCR model was perhaps the most important innovation in the model of Steibel et al (2009).

The experimental design might have involved additional "grouping factors" that are not directly related to the experimental treatments being studied but still might be responsible for a considerable proportion of variation and must be accounted for to achieve more accurate predictions. For example, the experiment might have involved repeated measurements of participating individuals, partitioning of the experimental subjects between several blocks (plots, tanks) for technical reasons, or measurements of all the effects of interest on different genotypes. The latter is the case in our coral example, where we used 8 coral colonies ("individuals") each split into four clonal fragments that were exposed to our experimental treatments. The grouping factors can be specified in the model as additional random factors; however, in contrast to the *sample* factor, these would be gene-specific since different genes might be affected by the grouping factors differently. In our case, we want to account for possible differences in baseline level of expression of each of our genes between 8 colonies, and we augment our model as follows:

ln(rate) ~ gene + gene:Condition + gene:Timepoint + gene:Timepoint:Condition + *sample* +
+ *gene:individual* .

Once again, the model is flexible in the number of grouping factors that could be included.

The two remaining terms that we still need to add are both error terms, accounting for the residual variation that remained unexplained. The first one is specified as a random factor and reflects the unexplained differences between biological replicates (samples). It makes sense to assume that this factor would be gene-specific, i.e., some genes will vary more than others among samples:

ln(rate) ~ gene + gene:Condition + gene:Timepoint + gene:Timepoint:Condition + *sample* +
+ *gene:individual* + *gene:sample* .

Finally, the remaining unexplained variation would be due to the differences between technical replicates, reflecting the precision of the qPCR instrument used. We follow Steibel et al (2009) who found that the model fit is typically improved when specifying this term as gene-specific:

ln(rate) ~ gene + gene:Condition + gene:Timepoint + gene:Timepoint:Condition + *sample* +
+ *gene:individual* + *gene:sample* + *gene:residual* . (2)

It is important to note that separating variances due to *gene:sample* from *gene:residual* is only possible when the dataset contains technical replicates (which, ideally, it should); otherwise the variances collapse into a single term *gene:residual*.

There is one last, but by no means the least, piece to the model formulation. We will assume additional variability due to Poisson process on top of all these sources of variation: the observed count is assumed to be a sample drawn from the Poisson distribution with the mean equal to the predicted count. This accounts for the increase in variance due to "shot noise" that might accompany down-regulations of low- and medium-abundant genes and allows for zero counts in the data. Note that the model does not assume that Poisson variation is the only source of residual variation: error terms terms within the model (2) capture any additional variation, and therefore account for the over-dispersion of the data.

Fitting the model: function `mcmc.qpcr()`

The model (2) may look quite complicated, but since many of its terms are universal to any qRT-PCR analysis, the syntax for the actual function call can be substantially simplified. To specify the model, construct appropriate priors, and fit it to the data without (at the moment) specifying any control genes, the function `mcmc.qpcr()` only requires the following:

```
mm=mcmc.qpcr(
      fixed="condition+timepoint+condition:timepoint",
      random="individual",
      data=dd
      )
```

As the function starts to run, it prints out constructed priors and MCMCglmm-aimed formulas for fixed and random effects (as a basic "sanity check"), and then proceeds to the MCMC chain reporting the number of iterations done and one useful value called "`Acceptance ratio for latent scores`". This value tells us whether the MCMC chain is still searching for a best-fit patch of parameters (smaller values at the beginning of the chain) or has reached it and is now sampling all the compatible parameter combinations (the values become more or less stable). By default, the chain does 12000 iterations ('`nitt=12000`') and discards the first 2000 of them ('`burnin=2000`') . If the plateau of the acceptance ratios is reached later than iteration 2000, we will need to adjust these parameters a bit. If, for example, the plateau is reached at iteration 4000, we will need to specify '`burnin=4000`' and also increase the total number of iterations proportionally (by 4000-2000 in our case) to obtain the same number of parameter samples, which comes to '`nitt=14000`'.

The object returned by the function ('`mm`') is of the MCMCglmm class, containing all the parameter values and deviances sampled during the progress of the MCMC chain. To appreciate the complexity of the actually fitted model, let's summarize it and see how many terms and interactions were involved. Below is the truncated result (omitting 11 genes out of 15), with annotations explaining the entries:

```
summary(mm)
  Iterations = 3001:12991
  Thinning interval  = 10
  Sample size  = 1000    # 1000 samples of parameter sets and deviances were stored

  DIC: 6479.916    # Deviance Information Criterion

  G-structure:  ~sample         # variance due to unequal template loading between samples

         post.mean l-95% CI u-95% CI eff.samp
  sample     1.493   0.7976    2.442     1000

                 ~idh(gene):individual    # gene-specific variance between individual corals

                    post.mean l-95% CI u-95% CI eff.samp
  actin.individual 0.0041456 8.862e-17 2.185e-02   103.85
  adk.individual   0.0006856 3.992e-17 3.379e-05   301.57
  ... # omitting entries for 11 genes
  spon2.individual 6.3975206 1.183e+00 1.521e+01  1000.00
  ubl3.individual  0.0571330 5.994e-17 2.996e-01    41.49

                 ~idh(gene):sample   # gene-specific variance between samples

              post.mean l-95% CI u-95% CI eff.samp
  actin.sample 0.2156528 9.914e-02  0.374336   512.84
  adk.sample   0.5445622 1.785e-01  0.961275   282.26
  ...
  spon2.sample 0.7653844 2.902e-01  1.421424   222.94
  ubl3.sample  0.2851004 6.560e-02  0.547365   113.78

   R-structure:  ~idh(gene):units     # gene-specific variance in measurement precision

             post.mean l-95% CI u-95% CI eff.samp
  actin.units 0.0650145 3.483e-02 0.098712  1000.00
```

```
adk.units   0.0710713 1.693e-02 0.145826   386.69
...
spon2.units 0.0292340 6.446e-03 0.061818   311.44
ubl3.units  0.0303894 6.487e-04 0.083863    79.43
Location effects: count ~ 0 + gene + gene:condition + gene:timepoint + gene:condition:timepoint

                                    post.mean l-95% CI u-95% CI eff.samp  pMCMC
# First, gene-specific intercepts, reflecting how abundant each gene is on average.
# These are of no interest to us.
geneactin                             9.35429  8.37311 10.16611  1134.68 <0.001 ***
geneadk                               2.33339  1.21747  3.28453  1100.97 <0.001 ***
...
genespon2                             3.40589  1.30645  5.43524  1000.00  0.002 **
geneubl3                              2.67307  1.72603  3.63866  1000.00 <0.001 ***
 # expression changes due to exposure to heat stress:
geneactin:conditionheat              -1.29628 -2.49431 -0.01555  1000.00  0.046 *
geneadk:conditionheat                 0.37930 -1.02631  1.83121  1000.00  0.574
...
genespon2:conditionheat               1.11408 -0.21414  2.69422  1000.00  0.124
geneubl3:conditionheat                0.51200 -0.70768  1.99122  1000.00  0.434
 # expression changes between two timepoints:
geneactin:timepointtwo               -0.96082 -2.15698  0.49938  1000.00  0.148
geneadk:timepointtwo                 -1.29126 -2.75501  0.17560   549.28  0.094 .
...
genespon2:timepointtwo               -1.57614 -2.98161  0.04681   669.22  0.044 *
geneubl3:timepointtwo                -1.94519 -3.27265 -0.62660   749.12  0.008 **
 # additional expression changes between two timepoints if a coral was heat-stressed at first:
geneactin:conditionheat:timepointtwo   1.85779 -0.08222  3.62050  1000.00  0.050 .
geneadk:conditionheat:timepointtwo     1.08814 -1.01211  2.94039  1000.00  0.302
...
genespon2:conditionheat:timepointtwo   0.29039 -2.00639  2.15243   775.15  0.780
geneubl3:conditionheat:timepointtwo    0.54378 -1.33804  2.31208   876.90  0.562
 ---
Signif. codes:  0 '***' 0.001 '**' 0.01 '*' 0.05 '.' 0.1 ' ' 1
```

The results may be ever so slightly different in each case of model fitting, because of the randomness in MCMC process. Note that the summary lists 95% credible intervals and MCMC-based p-values for each of the estimated parameters. A credible interval is a Bayesian analog of a confidence interval in frequentist statistics. Although a confidence interval may sound more familiar, the credible interval has a more intuitive interpretation, being the interval containing the true value of the parameter with a set probability (for example 0.95) given the data and the priors, whereas the confidence interval would be the range that includes the true parameter value in 95% of the independent re-runs of the experiment. The "location effects" (= fixed effects) will be of our primary interest. They are expressed as natural logarithms of fold-changes. qPCR practitioners might be more comfortable with the fold-changes on $\log_2$ scale (divide the listed values by `ln(2)`), since it would be just like the difference in number of qPCR cycles. We will later see how these values can be combined to calculate the difference between conditions of interest.

Also note that the summary for random effects does not show point-estimates (for example, for `~idh(gene):individual`, there is no value giving the expression of actin in the individual `s1`), only the variances attributable to each gene. Although it is possible to make the function save these estimates, they are of little practical value.

Diagnostic plots

There are three main criteria that can tell us whether our linear modeling approach is valid in application to the dataset at hand. First, the residuals of the model should not show a trend depending on the predicted value; if this is violated, then the data are not actually linear. Second, the size of the residuals should not change depending on the predicted value. This is the test for equal variances across the modeled range (homoscedastisity). Third, the residuals should be approximately normally distributed. For intricate statistical procedures such as linear mixed modeling, these assumptions are best tested graphically using three diagnostic plots: residuals plotted against predicted value to verify linearity, "scale-location" plot of the square root of absolute value of the residuals (i.e., the size of residuals) to test for homoscedasticity, and quantile-quantile plot of residuals against normal distribution to test for normality. The latter is simply a plot of ranked standardized (divided by their standard deviation) residuals against ranked normally-distributed variable, which is simulated. In our case, the criteria should apply to the lognormal residuals e_{gijk} from formula [2] (remember that we also have Poisson residuals on top of these). The MCMC.qpcr package includes a function *diagnostic.mcmc* that produces these plots; however, we first need to run the model with two additional options (`pr=T,pl=T`) to be able to extract the residuals:

```
mmd=mcmc.qpcr(
      fixed="condition+timepoint+condition:timepoint",
      random="individual",
      data=dd,
      pr=T,
      pl=T
      )
```

Then we can see whether our data fit the linear model assumptions reasonably well (Fig.2):

```
diagnostic.mcmc(
      model=mmd,
      col="grey50",
      cex=0.8
      )
```

(the arguments and `'col'` and `'cex'` here are not essential, they adjust the color and the size of the points on the plots)

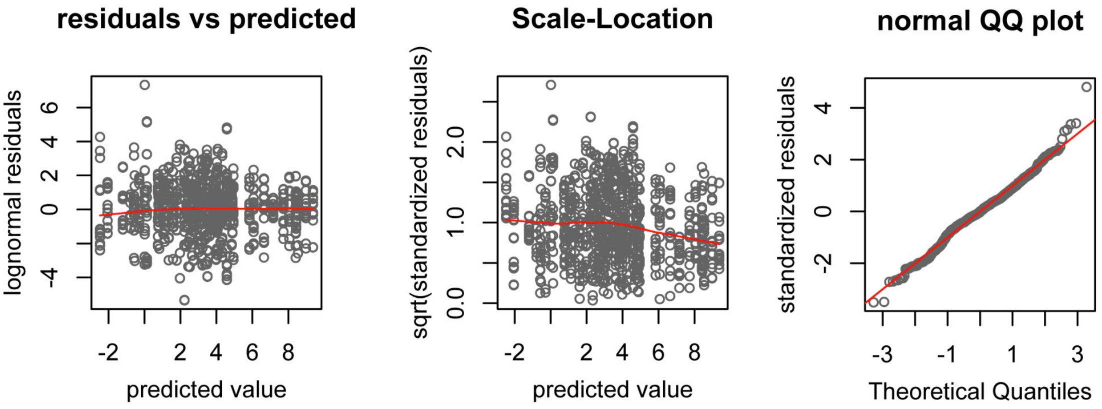

Figure 2. Diagnostic plots produced by the function `diagnostic.mcmc()`.

Extracting and visualizing results

One great advantage of the MCMC-based model fitting is that the estimates as well as credible intervals for any modeled effects (or their combinations) can be computed directly from the results of MCMC sampling.

Determining exactly which combination of factors corresponds to the contrasts of interest can be confusing for someone who is new to linear modeling, so I will briefly walk through the logic using our coral example. We want to know how coral genes are regulated in response to heat stress, and how they are then regulated after the coral is allowed to recover. So we want to find the combinations of fixed effects which would describe (i) the difference between stressed and unstressed coral on the first day, and (ii) the difference between recovering coral and its earlier stressed condition. Let's call the fist contrast "stress", and the second one "recovery". To see which combinations of effects describe these contrasts, it is also important to remember that all the effects listed in our model's summary are differences with respect to one particular combination of factor levels. Which one? The one that does not appear in the list of fixed effects in the summary. Conveniently, it seems to be the control condition at timepoint one; R chose it simply because 'control' and 'one' come alphabetically before 'heat' and 'two' (generally, any combination of factor levels can be specified as a reference using the generic `relevel()` function). Therefore, the "stress" contrast directly corresponds to '`conditionheat`' effect. The mean expression change for any particular gene, for example, `actin`, due to "stress" is thus given by the mean of the MCMC sample of the `geneactin:conditionheat` parameter, while its 95% credible interval is the range excluding top and bottom 2.5% of all sampled values. The means and credible intervals for such directly reportable effects are given in the model's summary:

```
…
                          post.mean l-95% CI u-95% CI eff.samp  pMCMC
geneactin:conditionheat     -1.29628 -2.49431 -0.01555  1000.00  0.046 *
```

'`pMCMC`' here is a two-tailed p-value, which is twice the fraction of all sampled values (out of '`eff.samp`', `1000`) that cross zero with respect to the mean. Note that, under this model, the actin down-regulation due to stress is significant at the 0.05 level.

"Recovery" is a trickier contrast: it is a compound effect, involving a combination of several directly reported effects. To compute it, remember that the expression of a gene in a stressed coral on the day of stress is, as we just discussed,

$$E_S = \texttt{[reference condition]} + \texttt{conditionheat} \ ,$$

while expression of a gene in a previously stressed coral on the day of recovery (timepoint 'two') is

$$E_R = \texttt{[reference condition]} + \texttt{conditionheat} + \texttt{timepointtwo} + \texttt{conditionheat:timepointtwo} \ .$$

Therefore, the difference in expression between these two conditions, representing gene regulation due to recovery from stress, is

$$E_R - E_S = \texttt{timepointtwo} + \texttt{conditionheat:timepointtwo} \ .$$

So, to compute the "recovery" effect for a gene, we have to sum up its '`timepointtwo`' and '`conditionheat:timepointtwo`' effects within each sampled iteration. These sums will then constitute a new MCMC sample for which the mean and credible interval can be computed in the same manner as for the directly reported effects.

Manually extracting and manipulating gene-specific effects from MCMC results can be tedious, to say the least. Function `HPDplot()` has been developed to streamline this processes and plot the results. It also converts the estimates from natural logarithm to $\log_2$.

The following will generate two separate point-and-whiskers plots for the "stress" and "recovery" effects across all genes (Fig. 3):

```
HPDplot(
      model=mm,
      factors="conditionheat",
      main="stress"
      )
HPDplot(
      model=mm,
      factors=c(
            "timepointtwo",
            "conditionheat:timepointtwo"
            ),
      main="recovery"
      )
```

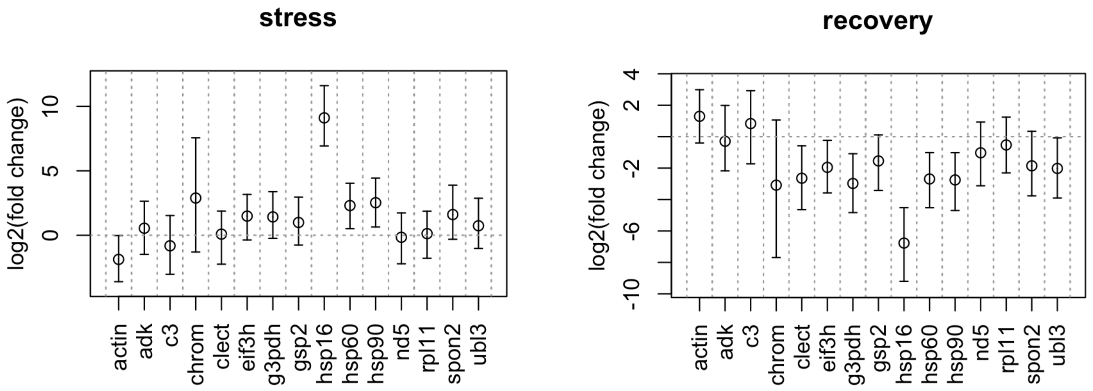

Figure 3. Fixed effects of stress and recovery according to the naïve model (no control genes specified). The points are posterior means, the whiskers denote 95% credible intervals. The figure is plotted with `HPDplot()`.

The argument '`factors`' here specifies the factors which need to be plotted. If more than one factor is listed, such as `c("timepointtwo","conditionheat:timepointtwo")`, these factors will be summed up – this is how compound effects, such as our "recovery", can be calculated. The argument "`main`" simply gives a title to the plot.

The fixed effects math goes just one step further. In models with fixed factors with more than two levels, to perform Tukey-style pairwise comparisons we might wish to subtract one type of effect from another, or, in a more general case, subtract one sum of effects from another sum of effects. To do this, the function `HPDplot()` can be provided with an additional argument, '`factors2`', which will specify the sum of effects to be subtracted from the one specified by '`factors`'.

Visually, it would be easier to compare the "stress" changes to "recovery changes if they were plotted on the same plot. This can be done with just a couple extra arguments (Fig. 4):

```
HPDplot(
      model=mm,
```

```
        factors="conditionheat",
        main="stress / recovery",
        jitter=-0.15,
        ylim=c(-10,12)
        )
HPDpoints(
        model=mm,
        factors=c(
                "timepointtwo",
                "conditionheat:timepointtwo"
                ),
        jitter=0.15,
        col="orange2",
        pch=17
        )
```

Note that the second function call is now to '`HPDpoints`', which adds to an existing plot rather than creates a new one. The parameter '`jitter`' shifts the dot-whiskers to the left if negative and to the right if positive, so the combined graphs would not overlap. '`ylim`' in the call to '`HPDplot`' sets the y-axis range, to make sure both graphs will fit. Finally, '`pch`' in the second call changes the type of symbol (see http://www.endmemo.com/program/R/pchsymbols.php for the chart of what's available) and '`col`' changes the color of the added graph (R colors: http://www.stat.columbia.edu/~tzheng/files/Rcolor.pdf).

A finishing touch is to add legend to the plot:

```
legend(11,12,"stress",lty=1,pch=1,bty="n")
legend(11,10,"recovery",lty=1,pch=17,col="orange2",bty="n")
```

Try `?legend` to see details about this basic R function. Briefly, it needs to be told the coordinates on the plot where to put the label (the first two numbers are x,y), type of line (if any), type of symbol (if any), color (black by default), and whether it should be in the box.

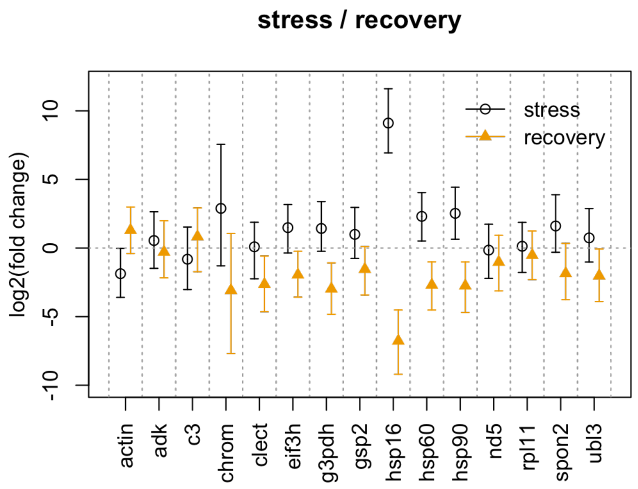

Figure 4. Fixed effects of stress and recovery according to naïve model (no control genes specified). The points are posterior means, the whiskers (left) or dashed lines (right) denote 95% credible intervals. The figure is plotted with `HPDplot()`, `HPDpoints()`, and `legend()`.

An alternative representation of the results is to plot them gene by gene for all conditions of interest. The function `HPDplotBygene()` does that, but first we need to create a 'list' object describing what are the

conditions we are looking to plot and which factor combinations they correspond to. Similarly to `HPDplot()` function, each condition can be described by two groups of fixed effects, '`factors`' and '`factors2`'. The effects within each group will be summed up, and then the sum for '`factors2`' will be subtracted from the sum for '`factors`'. In the vast majority of cases, a single group of effects ('`factors`') would be sufficient to describe conditions of interest. The groups may include the gene-specific intercept (which should be denoted by '0' among '`factors`'), corresponding to the gene expression level under 'reference' condition (see page 10).

First, we create the condition-defining list (review page 10 if you are still unsure how different conditions are described by sums of fixed effects):

```
cds=list(
   control=list(factors=0), # this is the gene-specific intercept, i.e., our reference condition
   stress=list(factors=c(0,"conditionheat")),
   recovery=list(factors=c(0,"conditionheat","timepointtwo","conditionheat:timepointtwo"))
   )
```

Now we can plot abundances of any gene under these conditions. Let's plot three most responsive genes, `actin`, `hps60` and `hsp16`. We will do it gene by gene, creating a plot panel with y-limits when plotting the first gene and then adding more graphs to the same plot, jittering them a little so they don't overlap:

```
HPDplotBygene(
      model=mm,
      gene="actin",
      conditions=cds,
      col="cyan3",
      jitter=-0.15,
      ylim=c(-3.5,15),
      pval="z"
      )
HPDplotBygene(
      model=mm,
      gene="hsp60",
      conditions=cds,
      newplot=FALSE,
      col="grey50",
      pval="z"
      )
HPDplotBygene(
      model=mm,
      gene="hsp16",
      conditions=cds,
      newplot=FALSE,
      col="coral",
      jitter=0.15,
      pval="z"
      )
```

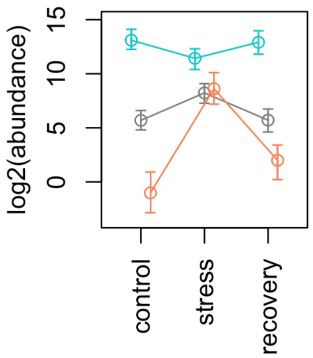

Figure 5. Abundances of `actin` (blue), `hps60` (grey) and `hsp16` (red) across experimental conditions, plotted using `HPDplotBygene()`. The whiskers denote 95% credible intervals.

Note that in addition to the plot (Fig. 5), the function `HPDplotBygene` outputs the matrix of mean pairwise differences (log2-transformed fold changes, think of them as the difference in number of PCR cycles) and the matrix of pairwise *p*-values, like shown here for the last call to `HPDplotBygene` (for `hsp16`):

```
$mean.pairwise.differences
         control   stress  recovery
control        0 6.678398  2.089490
stress         0 0.000000 -4.588908
recovery       0 0.000000  0.000000
```

```
$pvalues
        control stress     recovery
control       0      0 9.056390e-03
stress        0      0 3.904765e-11
recovery      0      0 0.000000e+00
```

Only the top triangles of the matrices are meaningful.

The p-values listed in this case are based on Bayesian z-score by specifying `pval="z"` among arguments, to approximate very small *p*-values from the limited MCMC sample size. See "Estimating statistical significance" section below for details on that.

If the experiment contains more than one grouping factor, function `HPDplotBygeneBygroup()` can plot up to three different groups on the same plot. For example, in another coral-related experiment that we recently performed, we heat-stressed corals from two different populations, 'offshore' and 'inshore'. Fig. 6 shows the plot produced by `HPDplotBygeneBygroup()` for one of the genes that we assayed (*coll*, collagen), split by the population of origin, across three coral conditions: normal, pale and bleached. The function takes two or three lists of conditions that define the groups to be plotted (note: the following commands will not work with the demo dataset):

```
inshore=list(
       normal=list(factors=0),
       pale=list(factors=c(0,"statuspale")),
       bleached=list(factors=c(0,"statusbleach"))
       )
offshore=list(
       normal=list(factors=c(0,"originoffshore")),
       pale=list(factors=c(0,"statuspale","originoffshore","originoffshore:statuspale")),
       bleached=list(factors=c(0,"statusbleach","originoffshore","originoffshore:statusbleach"))
       )

HPDplotBygeneBygroup(
       model=mm,
       gene="coll",
       group1=inshore,
       group2=offshore
       )
```

Incorporating information about control genes

Now it is time to remember that all the results we have seen thus far were obtained without telling the model which genes are expected to be stable. This is actually not required for the model to work correctly, even when there are only a few genes with very unbalanced pattern of changes (Matz, Wright, and Scott, 2013). This highlights the advantage of linear mixed model analysis, which makes the model "self-normalizing" by correctly inferring the variation due to unequal template loading, at least as long as the number of biological samples in the experiment is relatively large. Still, incorporating control genes into the analysis is a venerable tradition in qRT-PCR, and indeed this information helps to increase the power (i.e., narrow

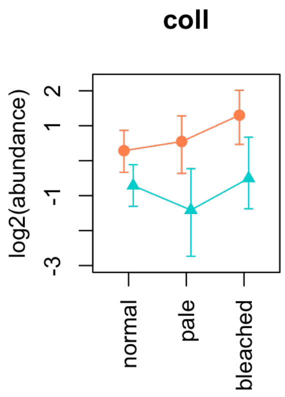

Figure 6. Relative abundances of *coll* across conditions in inshore (red circles) and offshore (blue triangles) corals. The whiskers denote 95% credible intervals. Plotted using `HPDplotBygeneBygroup()`.

down credible intervals) and might adjust the point estimates slightly, as we will see. More importantly, stable genes can be used to make sure that the model behaves reasonably: they should appear more or less unchanged even if the model is fitted without using them as priors, as we have done before in this tutorial.

The three control genes that were used in the published analysis of the `coral.stress` dataset (Kenkel et al. 2011) were `rpl11`, `nd5`, and `eif3h`. Looking at the plots of fixed effects from the 'naïve' model (Fig. 4), we can see that `nd5` and `rpl11` indeed appear more or less stable, while `eif3h` tends to come up under stress and down during recovery. We therefore choose to use only `rpl11` and `nd5` as control genes. Generally, the naïve model is a good way to identify potential control genes in the first place.

It must be emphasized, however, that inferring control genes from an experimental dataset and then using them to analyze the same dataset may lead to erroneous inference due to circularity. Ideally, control genes should be selected based on an independent experiment or other supporting data. If there is no such prior information, at the very least an alternative method of control gene selection should be used, such as non-parametric geNorm (Vandesompele et al, 2002). The MCMC.qpcr package includes a function `cq2genorm`, which reformates the data into log-transformed relative expression values and outputs the data in the form that may be directly fed into `selectHKgenes` function of SLqPCR package, which is the R implementation of geNorm algorithm. Try ?`cq2genorm` for more details.

To fit the 'informed' model using these two control genes, we must tell the model not only what are our controls, but also how stable we expect them to be. We actually don't have to assume that the specified controls are fully stable, which is a particularly nice touch in Bayesian analysis. In the syntax of `mcmc.qpcr()` function, the control genes' stability is specified using `m.fix` and `v.fix` parameters, which correspond to the average allowed fold change in response to fixed and random factors, respectively. The default values of `m.fix` is 1.2 (and of `v.fix` is NULL, no fixation) but it can be increased to reflect greater uncertainty about the stability of control genes, or lowered all the way down to 1, which would mean that the control genes are required to be perfectly stable (hardly ever a realistic assumption, Thellin et al. 1999). The parameter that has the most effect on the estimates is `m.fix`, we are only keeping `v.fix` because it seems reasonable and does not hurt.

Let's fit the 'informed' model with `rpl11` and `nd5` as control genes and `m.fix=1.2` to the `dd` data and compare the results to the 'naïve' model. But first, we will re-fit the naïve model with the arguments that tell the model what the control genes are but do not let the model use them ('`include=0`'). The estimates will not change, but the order of the genes in the output will be the same as in the informed model, so their results can be compared on the same plot.

```
naive=mcmc.qpcr(
      fixed="condition+timepoint+condition:timepoint",
      random="individual",
      data=dd,
      controls=c("rpl11","nd5"),
      include=0
      )
informed=mcmc.qpcr(
      fixed="condition+timepoint+condition:timepoint",
      random="individual",
      data=dd,
      controls=c("rpl11","nd5"),
      m.fix=1.2
      )
```

Plotting "stress" effects (Fig. 7, left panel):

```
HPDplot(
      model=naive,
```

```
        factors="conditionheat",
        main="stress",
        hpdtype="l"
        )
HPDpoints(
        model=informed,
        factors="conditionheat",
        hpdtype="l",
        col="coral"
        )
```

Plotting "recovery" effects (Fig. 7, right panel):

```
HPDplot(
        model=naive,
        factors=c("timepointtwo","conditionheat:timepointtwo"),
        main="recovery",
        hpdtype="l"
        )
HPDpoints(
        model=informed,
        factors=c("timepointtwo","conditionheat:timepointtwo"),
        hpdtype="l",
        col="coral"
        )
```

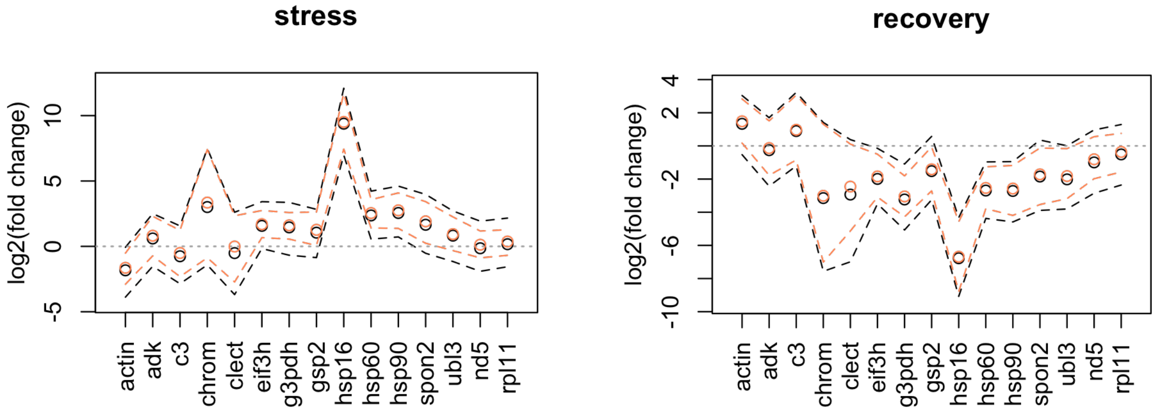

Figure 7. Comparison of the informed (orange) and naïve (black) models. The informed model used two control genes, `rpl11` and `nd5`, with `m.fix` (average allowed fold-change) =`1.2`; the naïve model did not use any control gene information. The points are posterior means, the dashed lines denote 95% credible intervals. The figure is plotted with `HPDplot()` and `HPDpoints()` with the option `'hpdtype="l"'`.

This time, to better evaluate the changes in the width of credible intervals, we used the option 'hpdtype="l"' for `HPDplot()` and `HPDpoints()`. It makes the functions draw dashed lines across upper and lower 95% credible interval limits of all genes.

We can see (Fig. 7) that informing the model about control genes and allowing them to be regulated 1.2 fold on average has virtually no effect on the point estimates, but noticeably narrows the credible intervals, so the informed model is more powerful. In fact, almost the same increase in power is already provided by including a single control gene.

Estimating statistical significance

Significant (at the alpha = 0.05) fixed effects are the ones in which the 95% credible interval does not include zero, such as, for example, `hsp16`, `hsp70`, or `hsp90` on Fig. 4. However, we are typically performing multiple comparisons of this kind within a qRT-PCR experiment, and so the resulting significances must be corrected accordingly. The solution provided within the MCMC.qpcr package is to calculate p-values for all the effects of potential interest (i.e., all of those which were given a chance to become significant) and correct them for multiple testing using one of the established methods. The p-values can be obtained in two ways. For large MCMC samples it is practical to use a Bayesian two-sided p-value, which is twice the fraction of all sampled values that crosses zero with respect to the mean. The lowest p-value that can be thus obtained is 2 divided by the size of MCMC sample; so for the default set of MCMC chain parameters that results in 1,000 samples it cannot be lower than 0.002. To approximate lower p-values based on a limited MCMC sample size, we assume that the posterior distributions of a parameter can be represented by a normal distribution, calculate a Bayesian z-score (the mean of the posterior divided by its standard deviation) and perform a standard z-test to derive a two-tailed p-value. The z-score based p-values agree with MCMC-based ones very well and are distributed uniformly under the null hypothesis (Matz, Wright, and Scott, 2013).

To obtain point-estimates of posterior modes, means, 95% credible intervals, and uncorrected p-values for fixed effects from our informed model, we will run the function `HPDplot()` with a flag ‘`plot=FALSE`’:

```
stress=HPDplot(
      model=informed,
      factors="conditionheat",
      plot=FALSE
      )
stress
                                 mode        mean       lower      upper       pval.z pval.mcmc
   geneactin:conditionheat -1.5269197 -1.63992250 -2.89236066 -0.5189583 6.801355e-03     0.006
   geneadk:conditionheat    0.8814979  0.81502759 -0.72771887  2.3017463 2.906479e-01     0.270
   ...
   genehsp60:conditionheat  2.7582144  2.54611536  1.40805668  3.5734072 3.178015e-06     0.002
   genehsp90:conditionheat  2.6241394  2.75244674  1.37413737  4.0876553 5.664635e-05     0.002
   ...
   genend5:conditionheat    0.1592772  0.12640394 -0.87753846  1.1750965 8.113947e-01     0.814
   generpl11:conditionheat  0.3852459  0.36824375 -0.68370549  1.2741158 4.703683e-01     0.494
```

This was for the effects of “stress”, let’s obtain the “recovery” estimates, too:

```
recovery=HPDplot(
      model=informed,
      factors=c("timepointtwo","conditionheat:timepointtwo"),
      plot=FALSE
      )
recovery
                           mode       mean      lower        upper       pval.z pval.mcmc
   geneactin ...     1.52610996  1.4851758  0.1752831  2.83182731 3.190544e-02     0.024
   geneadk ...       0.06651484 -0.1314239 -1.7749815  1.50854783 8.766451e-01     0.898
   ...
   genehsp60 ...    -2.35357919 -2.5368612 -3.7839781 -1.25969779 8.798320e-05     0.002
   genehsp90 ...    -2.36500390 -2.5724855 -4.1916809 -1.17988543 8.776660e-04     0.002
   ...
   genend5 ...      -0.79335215 -0.8088653 -1.9794449  0.55651985 2.186604e-01     0.208
   generpl11 ...    -0.35166553 -0.3625902 -1.5735637  0.75848378 5.511591e-01     0.550
```

(The names of the effects in this case have been truncated to fit the table into page.)

Note that the control genes `("rpl11","nd5")` are still there, appearing in the end of each list.

Now, we will concatenate these results into a single table `all.effects` and correct the p-values for multiple testing using Benjamini-Hochberg method (Benjamini, Hochberg 1995), while disregarding the entries corresponding to the control genes since these are not among our effects of interest:

```
all.effects=rbind(stress,recovery)
padj.qpcr(all.effects, controls=c("rpl11","nd5"))
                         pval.z pval.mcmc       padj.z  padj.mcmc
     # stress:
  geneactin ...   6.801355e-03     0.006 1.607593e-02 0.014181818
  geneadk ...     2.906479e-01     0.270 3.434930e-01 0.319090909
  ...
  genehsp60 ...   3.178015e-06     0.002 2.065710e-05 0.005777778
  genehsp90 ...   5.664635e-05     0.002 2.945610e-04 0.005777778
  ...
  genend5 ...               NA        NA           NA          NA
  generpl11 ...             NA        NA           NA          NA

     # recovery:
  geneactin ...   3.190544e-02     0.024 5.925296e-02 0.044571429
  geneadk ...     8.766451e-01     0.898 9.117109e-01 0.933920000
  ...
  genend5 ...               NA        NA           NA          NA
  generpl11 ...             NA        NA           NA          NA
```

The output of `padj.qpcr()` actually has full names of effects and contains all the original columns (including mean, mode, and credible interval). Now the p-values for the control genes are dropped from consideration.

Lognormal model for higher-abundance data

In datasets where all the targets always remain relatively abundant, there will be little or no Poisson-induced variation. In such cases, it is possible to perform the Bayesian analysis described here based on the lognormal model without the Poisson component, thus sidestepping the need to make assumptions concerning Cq1. MCMC.qpcr package implements this model as function `mcmc.qpcr.lognormal`. The data for this function must be prepared using the function `cq2log`, which is analogous to `cq2counts` but converts the Cq values into natural logarithms of relative abundances (*Ra*) while correcting for the efficiency of amplification using the following formula (Steibel et al, 2009; Kenkel et al, 2011):

$$Ra = - Cq \cdot \ln (E) \quad . \tag{4}$$

These values are processed using the same model but lacking the Poisson component, with the same possibilities for specifying control genes as priors as well as for downstream statistical analysis and visualization. The model is expected to generate the same or very similar results as the Poisson-lognormal model for datasets that do not have many Cq values above 30. It is important to remember that, since the lognormal model cannot adequately deal with the instances of no amplification, such datapoints must be excluded from the analysis or replaced by some arbitrarily high Cq value (for example, 38), which may bias the inference if such instances are frequent – so use the Poisson-lognormal model for such cases.

The "classic" model

One of the key assumptions of the MCMC.qpcr model is that the template loadings, which reflect the amount and quality of RNA across samples, are drawn from the same gaussian distribution irrespective of

the condition. In other words, even though RNA quantity and quality is allowed to vary between samples, it is assumed to vary in the same way under all conditions. If for some conditions the RNA samples are consistently of lower concentration and/or poorer quality, the model will infer down-regulation of all genes under these conditions. Accounting for such a bias would unavoidably require falling back to reliance on control genes for normalization. For such cases, and assuming that the targets remain relatively abundant not worry about shot noise (the majority of the Cq values are below 30), we implemented the "classic" approach that starts with the log-transformed relative abundances (*Ra*, formula 4) produced by the `cq2log()` function, performs multigene normalization (Vandesompele et al, 2002), and then analyzes all the genes jointly within a single linear mixed model fitted by MCMC. We call this model "classic" since it is mostly based on earlier developments and lacks the main advancements implemented within the MCMC.qpcr package, such as the use of generalized linear modeling to account for higher variance of low-abundant targets and the possibility to analyze the data without using control genes. The one innovation the "classic" model offers, however, is a single-model rather than gene-by-gene analysis, which boosts the power considerably since the model draws evidence from all genes simultaneously. The "classic" model uses the same formula (2), only omitting the sample-specific variance and Poisson components. The function `mcmc.qpcr.classic`(), implementing this model, uses default uninformative priors of the `MCMCglmm` function, which results in estimates corresponding to maximum likelihood analysis.

To analyze the coral stress data using the "classic" model, we first must create a log-transformed dataset (this command uses very similar syntax as `cq2counts`, see page 4):

```
dl=cq2log(
      data=coral.stress,
      genecols=c(5:19),   # where the Cq data are in the data table
      condcols=c(1:4),    # which columns contain factors
      effic=amp.eff,
      noamp=38
      )

   There were 11 warnings (use warnings() to see them)
```

The warnings are produced because the instances on no-amplification, coded as -1, were encountered in the data. They were replaced by the value specified as '`noamp`' argument: 38 (the default) is an arbitrarily low, but not too low, value corresponding slightly less than one molecule per amplification trial. Use '`noamp=NA`' to disregard such instances completely. Technically, such low-abundance datasets should be analyzed using Poisson-lognormal model.

Let's fit the "classic" model with `pl=T,pr=T` options for us to be able to plot the diagnostic plots using `diagnostic.mcmc()`:

```
classic=mcmc.qpcr.classic(
      fixed="condition+timepoint+condition:timepoint",
      random="individual",
      data=dl,
      controls=c("nd5","rpl11"),
      pr=T,
      pl=T
      )
diagnostic.mcmc(
      model=classic,
      col="grey50",
      cex=0.8
      )
```

The arguments and `'col'` and `'cex'` here are not essential, they adjust the color and the size of the points on the plots.

The plots (Fig. 8) show that the "classic" model satisfies the linearity ("residuals vs predicted" plot) and homoscedasticity ("Scale-Location" plot) criteria reasonably well, although its residuals do not fully conform to the normal distribution ("normal Q-Q plot"). This is most likely the result of Poisson variation existing in the data as well as no-amplification instances, since the residuals of the Poisson-lognormal model were much closer to normal (see Fig. 2).

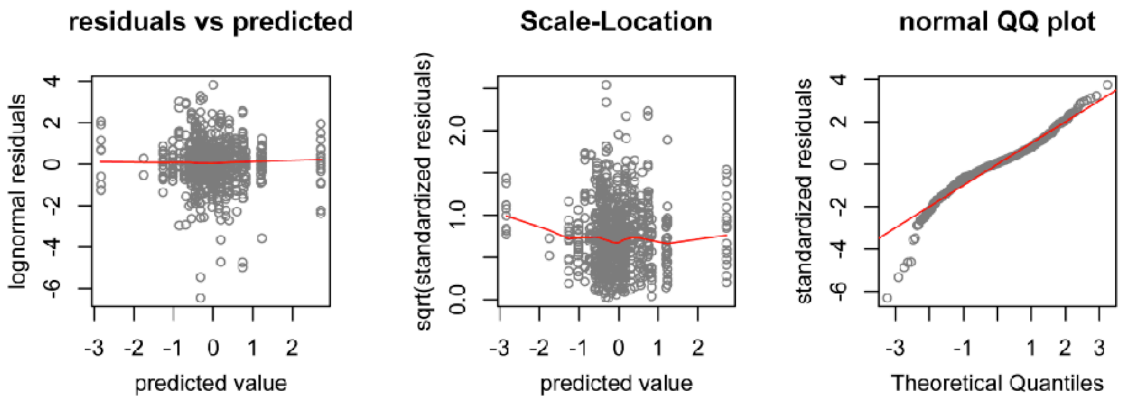

Figure 8. Diagnostic plots for the "classic" (normalizing) model (compare to Fig. 2)

Let's compare the inference of the "classic" model to our full-Bayesian informed model (Fig. 9):

```
HPDplot(
      model=informed,
      factors="conditionheat",
      pch=19,
      col="cyan3",
      jitter=-0.15,
      main="stress"
      )
HPDpoints(
      model=classic,
      factors="conditionheat",
      pch=19,
      col="coral",
      jitter=0.15,
      )
legend(11,10.5,"informed",bty="n",pch=19,col="cyan3")
legend(11,9,"\"classic\"",bty="n",pch=19,col="coral")
```

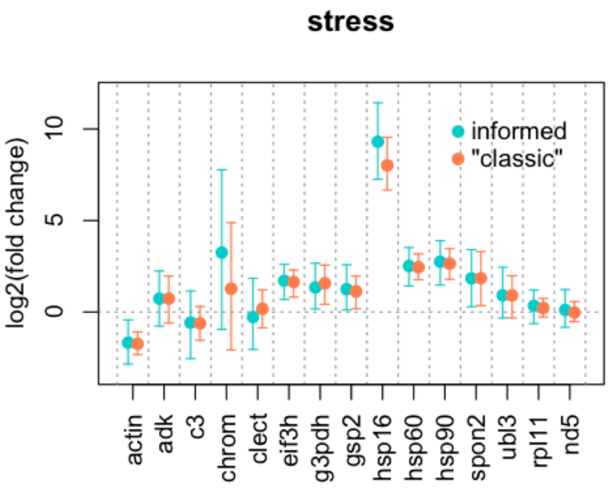

Figure 9. Comparison of the predictions of the informed model with two control genes and "classic" (normalizing) model. Credible intervals of the "classic" model are typically narrower, so the model is more powerful as long as the average stability of the control genes can be fully trusted.

See pagecisc 12 for explanations of the 'beautifying' tricks used here, such as options `pch`, `col`, and `jitter`, and the `legend()` function.

We can see that the "classic" model infers virtually the same fold-changes as the full-Bayesian informed model, but does it with higher confidence since the credible intervals are typically narrower. The "classic" model therefore represents a powerful alternative to the full Bayesian analysis when the expression levels are not too low. It is important to remember, however, that the high power of the "classic" model comes at the cost of making strong assumptions about control gene stability, so the Bayesian models (informed and especially naïve) represent a more conservative approach. Still, the "classic" model might be the only option in the cases when the quantity and/or quality of the RNA samples varies considerably and systematically across experimental conditions.

References

Benjamini, Y, Y Hochberg. 1995. Controlling the False Discovery Rate - a Practical and Powerful Approach to Multiple Testing. Journal of the Royal Statistical Society Series B-Methodological 57:289-300.

Clayton, D, J Kaldor. 1987. Empirical Bayes Estimates of Age-Standardized Relative Risks for Use in Disease Mapping. Biometrics 43:671-681.

Elston, DA, R Moss, T Boulinier, C Arrowsmith, X Lambin. 2001. Analysis of aggregation, a worked example: numbers of ticks on red grouse chicks. Parasitology 122:563-569.

Hadfield, JD. 2010. MCMC Methods for Multi-Response Generalized Linear Mixed Models: The MCMCglmm R Package. Journal of Statistical Software 33:1-22.

Kenkel, CD, G Aglyamova, A Alamaru, et al. 2011. Development of gene expression markers of acute heat-light stress in reef-building corals of the genus Porites. Plos One 6:e26914.

Kruuk, LEB. 2004. Estimating genetic parameters in natural populations using the 'animal model'. Philosophical Transactions of the Royal Society of London Series B-Biological Sciences 359:873-890.

Luu-The, V, N Paquet, E Calvo, J Cumps. 2005. Improved real-time RT-PCR method for high-throughput measurements using second derivative calculation and double correction. Biotechniques 38:287-293.

Matz, MV., Wright, R.M, and Scott, J.G. 2013. No control genes required: Bayesian analysis of qRT-PCR data. PLoS ONE.

Pfaffl, MW. 2001. A new mathematical model for relative quantification in real-time RT-PCR. Nucleic Acids Research 29:e45.

Poletto, R, JP Steibel, JM Siegford, AJ Zanella. 2006. Effects of early weaning and social isolation on the expression of glucocorticoid and mineralocorticoid receptor and 11β-hydroxysteroid dehydrogenase 1 and 2 mRNAs in the frontal cortex and hippocampus of piglets. Brain Res 1067:36-42.

Steibel, JP, R Poletto, PM Coussens, GJ Rosa. 2009. A powerful and flexible linear mixed model framework for the analysis of relative quantification RT-PCR data. Genomics 94:146-152.

Thellin, O, W Zorzi, B Lakaye, B De Borman, B Coumans, G Hennen, T Grisar, A Igout, E Heinen. 1999. Housekeeping genes as internal standards: use and limits. J Biotechnol 75:291-295.

Vandesompele, J, K De Preter, F Pattyn, B Poppe, N Van Roy, A De Paepe, F Speleman. 2002. Accurate normalization of real-time quantitative RT-PCR data by geometric averaging of multiple internal control genes. Genome Biology 3:research0034.0031-0034.0011.
